# Integrative Analysis of Metabolome and Transcriptome Reveals the Mechanism of Color Formation in *Liriope spicata* Fruit

**DOI:** 10.3390/metabo12020144

**Published:** 2022-02-04

**Authors:** Sichen Gan, Gang Zheng, Shoukuo Zhu, Jieyu Qian, Lijun Liang

**Affiliations:** College of Landscape Architecture, Zhejiang Agriculture & Forestry University, Hangzhou 311300, China; 2019105052020@stu.zafu.edu.cn (S.G.); zhengyj@zafu.edu.cn (G.Z.); shoukuozhu@stu.zafu.edu.cn (S.Z.); 2019105052025@stu.zafu.edu.cn (J.Q.)

**Keywords:** *Liriope spicata*, fruit skin, anthocyanins, metabolome, transcriptome

## Abstract

*Liriope spicata* is an important ornamental ground cover plant, with a fruit color that turns from green to black during the development and ripening stages. However, the material basis and regulatory mechanism of the color variation remains unclear. In this study, a total of 31 anthocyanins and 2 flavonols were identified from the skin of *L. spicata* fruit via integrative analysis on the metabolome and transcriptome of three developmental stages. The pigments of black/mature fruits are composed of five common anthocyanin compounds, of which Peonidin 3–*O*–rutinoside and Delphinidin 3–*O*–glucoside are the most differential metabolites for color conversion. Using dual-omics joint analysis, the mechanism of color formation was obtained as follows. The expression of structural genes including *4CL*, *F3H*, *F3′H*, *F3′5′H* and *UFGT* were activated due to the upregulation of transcription factor genes *MYB* and *bHLH*. As a result, a large amount of precursor substances for the synthesis of flavonoids accumulated. After glycosylation, stable pigments were generated which promoted the accumulation of anthocyanins and the formation of black skin.

## 1. Introduction

Anthocyanins belong to flavonoid compounds, generally sourced from secondary metabolites. Their basic structure contains two aromatic rings and one heterocyclic ring. Anthocyanins generally present different colors due to their functional groups, for example, Pelargonidin displays orange and Cyanidin and Peonidin show red/pink, while Delphinidin, Malvidin and Petunidin demonstrate blue/purple [1]. However, the color of the plant organ is not formed from single pigment, but from a variety of anthocyanin compounds. Various anthocyanins, which accumulate in the epidermic cells in different proportions, generally influence the final color of the organ. The increased ratio of Cyanidin/Pelargonidin in *Zea mays* has been reported to be the key reason for the purple tint in red grains [2]. Lou et al. found that the improved proportion of Cyanidin/Delphinidin led to the purple petals in Muscari [3]. The color of anthocyanins is also affected by cellular internal factors. *Pueraria Montana*, for instance, produced purple petals under an increased pH of flower vacuole, but remained stable in the structure of anthocyanin [4]. This phenomenon was also found in *Primula malacoides* [5]. In addition, colorless co-pigments such as flavonols and flavones, can combine with anthocyanin to form non-covalent complexes and, as a result, the hue will change. However, the content of co-pigments in dark purple/black plant organs is usually low [6,7].

It is well known that the pathway for anthocyanin synthesis is conserved, and this has been proven in various plants such as *Arabidopsis thaliana* [8], *Capsicum annuum* [9], *Pyrus* spp. [10] and *Malus × domestica* [11]. Anthocyanin is biosynthesized from phenylalanine, which is firstly converted into a precursor under the catalysis of enzymes encoded by a series of structural genes, including *phenylalanine ammonia lyase* (*PAL*), *cinnamate 4–hydroxylase* (*C4H*), *4–coumaric acid: CoA ligase* (*4CL*), *chalcone synthase* (*CHS*), *chalcone isomerase* (*CHI*), *flavanone 3–hydroxylase* (*F3H*), *flavonoid 3′–hydroxylase* (*F3′H*), and *flavonoid 3′5′–hydroxylase* (*F3′5′H*). Subsequently, colored anthocyanin is formed with the catalyzation of *dihydroflavonol–4–reductase* (*DFR*), *anthocyanidin synthase* (*ANS*) and *UDP–glucose: flavonoid 3–O–glucosyltransferase* (*UFGT*), etc. [11,12].

The accumulation of anthocyanin is also affected by transcription factors (TFs), which can regulate the expression of structural genes. The most common TFs are the ternary-protein complex (MBW) composed of DNA-binding R2R3-MYB transcription factor (MYB), basic helix-loop-helix (bHLH) transcription factor, and WD40 repeat protein (WD40). *WD40* is almost transcribed in all tissues and its expression level is hardly positively correlated with both the transcription level of structural genes and the content of anthocyanin [13,14,15]. However, *MYB* genes and *bHLH* genes have been proven to be specifically expressed in many anthocyanin-rich tissues [16,17,18,19]. The regulated roles of these two TFs in the MBW are disclosed as follows. MYB determines the activation and inhibition characteristics of the MBW complex while bHLH interacts with MYB and WD40. Xu et al. proved that *DCMYB6* was the key gene for the purple mutated carrot (*Daucus carota*), and allogenic overexpression of *DCMYB6* would induce the anthocyanins accumulation in both nutritional and reproductive tissues of *Arabidopsis thaliana,* and at the same time upregulate seven structural gene expressions [20]. In comparison, the overexpression of *PtMYB165* and *PtMYB194* in *Populus tremula × P. tremuloides* restrained several structural genes in the phenylalanine and flavonoid metabolism pathway, leading to the decreased contents of upstream products (phenolic glycoside and hydroxycinnamic acid ester) and downstream products (anthocyanidins and proanthocyanidins) [21]. *bHLH* genes have been reported to regulate the accumulation of anthocyanidins in many ways, for example, in *Paeonia suffruticosa, PsbHLH1* combines with the promoters of *PsDFR* and *PsANS* to control anthocyanin accumulation [22]. *DcbHLH5* in *Dracaena cambodiana* has also been confirmed to be capable of activating *CHS* and *CHI* for anthocyanin regulation [23].

*Liriope spicata*, a perennial herb plant, belongs to Liliaceae. Due to its strong shade tolerance, it is widely distributed in China, except for extremely cold and/or high-altitude areas [24]. The skin of the *L. spicata* fruit turns from green to black when ripening, and the duration for the ornamental period is as long as the whole winter. The scape takes longer than the leaves to observe and accordingly, *L. spicata* is accepted as a superior fruit plant in landscape application. Studies have revealed that black plant tissues are rich in anthocyanins [6,7,25,26,27] and the main metabolites in the black fruit skin of *L. platyphylla* are Petunidin–3–*O*–rutinoside and Malvidin–3–*O*–rutinoside [28]. However, few reports have addressed the mechanism for the color formation of the fruit skin of *L. spicata*. 

The integration of metabolome and transcriptome is considered as an effective method for analyzing the interactions between genes and metabolites. This joint analysis has been widely used in studying the color formation of Fragaria fruits [29], *Lonicera japonica* flowers [30], and the Cattley color differentiation of floral segments [31]. In this study, transcriptome and metabolome joint analyses were carried out on the skin of *L. spicata* fruits selected from three developmental stages, and a total of 31 anthocyanins and 2 flavonols were identified. Moreover, the expression profiles of genes related to the anthocyanin synthesis pathway were obtained by transcriptome. On these bases, the key genes for color transformation were screened using correlation analysis between genes and metabolites. This study not only elaborated the laws for anthocyanin accumulation in *L. spicata* fruits, but also revealed the molecular mechanism of fruit color transformation which the laid foundations for the color cultivation of ornamental fruits.

## 2. Results

### 2.1. Relationship between the Color Development and the Variation of Chlorophyll and Anthocyanin

**Figure 1:**
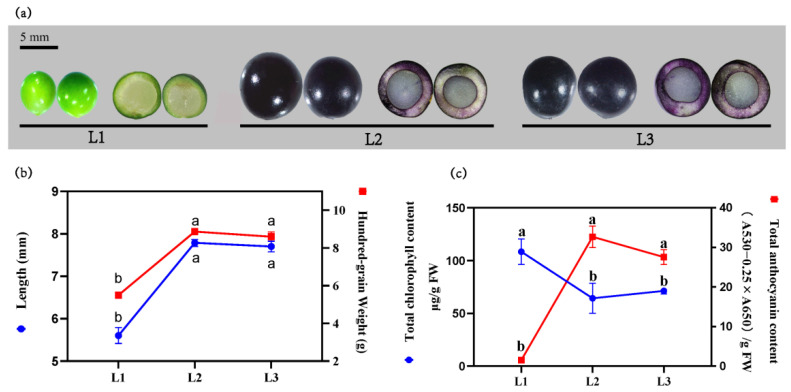
The dynamic variation of *L. spicata* fruits in morphology and pigment during three developmental stages. (**a**) The variation of fruit appearance, (**b**) length and hundred-grain weight, (**c**) TCC and TAC. Error bars represent ± SE of biological replicates (ten biological replicates × three stages, *n* = 30). Different lowercased letters in the same panel indicate statistical significance using Duncan’s test (*p* < 0.05).

The morphology of *L. spicata* fruit at the three stages are shown in Figure 1a. At the first stage (L1), the fruits displayed an immature, green color on the skin, with a transparent inner part. In stage two (L2) and stage three (L3), the fruits gradually grew mature, showing a darkening skin, but an unchanging transparent inner part. The variation of fruit color could be ascribed to the changes in pigments. At L1, the total content of chlorophyll (TCC) reached its highest, while the relative content of total anthocyanin (TAC) was not accumulated. At L2, the fruits expanded rapidly with the color completely turning into black. The hundred-grain weight (HGW) and dimension of the long axis (length) were 1.61-fold and 1.38-fold higher than that in L1, correspondingly. The TCC decreased significantly during L2 compared with L1, and conversely, the TAC increased significantly. L3 saw insignificant changes in the HGW, length and color of fruit in comparison with L2, and the TCC and TAC were also found to change slightly (*p* < 0.05) (Figure 1b,c). 

### 2.2. The Metabolites Profiles of L. spicata Fruits

**Figure 2:**
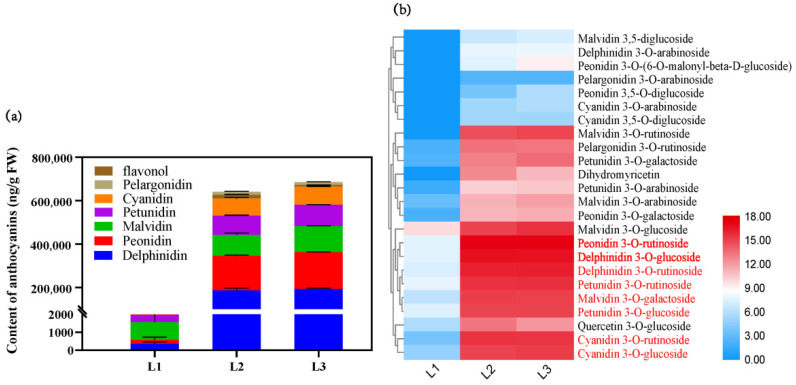
Metabolite analysis of *L. spicata* fruits in different stages. (**a**) The content variation of anthocyanins and flavonol in *L. spicata* fruits. Error bars represent ± SE of biological replicates (three biological replicates × three stages, *n* = 9). (**b**) The heat map of DAMs during three stages. Intensity value bars are shown on the right side of the heat map. Colors indicate the normalized intensity of DAMs, of which the red color represents high content and blue corresponds to low content.

A total of 33 flavonoids were obtained from the fruits of L. spicata, including 31 anthocyanins and 2 flavonols. The black skin of *L. spicata* was mainly composed of five anthocyanin types (Figure 2a, Supplementary Table S1), including Delphinidin and its *O*-methylated derivatives (Petunidin and Malvidin), and Cyanidin and its *O*-methylated derivatives (Peonidin). Five types of anthocyanins accounted for about 97.73% and 98.02% of the total amount of metabolites in L2 and L3, respectively. Delphinidin and Peonidin were the highest among the five anthocyanins. 3–*O*–glucoside and 3–*O*–rutinoside were the two dominant Delphinidin type. Peonidin 3–*O*–rutinoside accounted for nearly 95% of Peonidin-type in L2 and L3, and was even listed as the vast majority of total anthocyanin content in L2 and L3.

### 2.3. Differentially Accumulated Metabolites (DAMs) Analysis on the Fruit

The differentially accumulated metabolites (DAMs) of fruit skin were screened from every two adjacent stages during the fruit development (L1 to L2 and L2 to L3), and the thresholds were set as VIP ≥ 1, *p* < 0.05, Log_2_FC ≥ 1 or Log_2_FC ≤ −1. A total of 24 kinds of flavonoids were obtained, including 22 anthocyanins and 2 flavonols. Figure 2b presents a substantial increase in DAMs from L1 to L2 and stable DAMs from L2 to L3. Peonidin 3–*O*–glucoside was the only metabolite which had significant differences between L2 and L3, and the VIP was 1.16. The large amounts of DAMs in black skin appearing from L2 to L3 were Delphinidin 3–*O*–glucoside, Peonidin 3–*O*–rutinoside, Delphinidin 3–*O*–rutinoside, Petunidin 3–*O*–rutinoside, Malvidin 3–*O*–galactoside, Petunidin 3–*O*–glucoside, Cyanidin 3–*O*–rutinoside and Cyanidin 3–*O*–glucoside (Table S1). Among which, Peonidin 3–*O*–rutinoside and Delphinidin 3–*O*–glucoside were the most abundant anthocyanins, with VIP values at 1.05 and 1.02 corresponding to the stages L1 to L2 and L2 to L3.

Two flavonols named Quercetin 3–*O*–glucoside and Dihydromyricetin also increased significantly from L1 to L2, but their contents were much lower than that of anthocyanins, accounting for 2.66% and 1.03% of the DAMs in L2 and L3 (Table S1), respectively. 

### 2.4. Differential Expression of Flavonoid Structural Genes and Regulatory Genes (DEGs) Analysis on the Fruit

**Figure 3:**
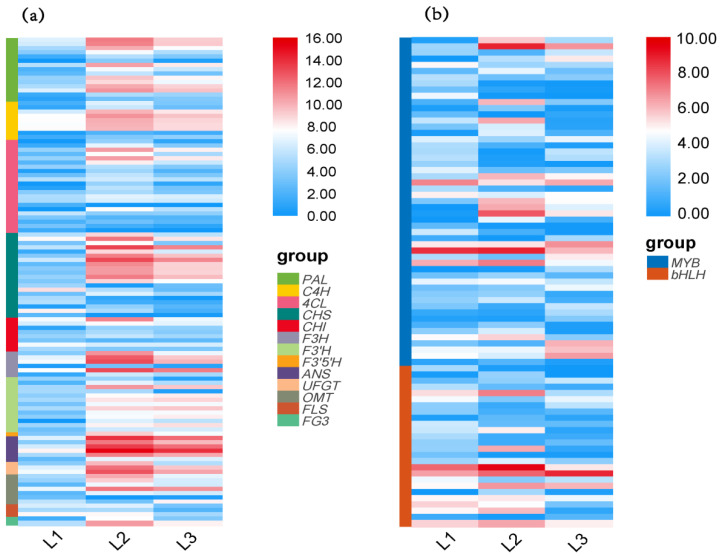
Heat map of DEGs. (**a**) The dynamic profile of differentially expressed structural genes and (**b**) regulation genes. Intensity value bars are shown on the right side of each heat map. Colors indicate the normalized intensity of DEGs. Red represents high expression and blue corresponds to low expression.

To further understand the mechanism of color formation of *L. spicata* fruits, the expression patterns of structural genes in the anthocyanin and flavonol pathways were analyzed. Based on KEGG, a total of 345 genes were found to be involved in the synthesis of anthocyanin and flavonol, and 184 of which were differentially expressed (Table 1, Supplementary Table S2). The genes annotated as *PAL, C4H, F3H, F3′H, F3′5′H, ANS, UFGT* and *F3G* were significantly upregulated at L2. Most of the genes in *4CL, CHS, CHI, OMT* and *FLS* families were upregulated at L2 except for a few downregulating, including five *4CL*, six *CHS*, three *CHI*, two *OMT* and one *FLS*. *ANSs* genes including Cluster 4331.95361, Cluster 4331.95047, Cluster 4331.91922 displayed the highest expression level in L2, about 161.80-fold, 158.45-fold and 149.95-fold higher than in L1, correspondingly. Most structural genes were downregulated in L3 (Figure 3a, Supplementary Table S2). 

**Figure 4:**
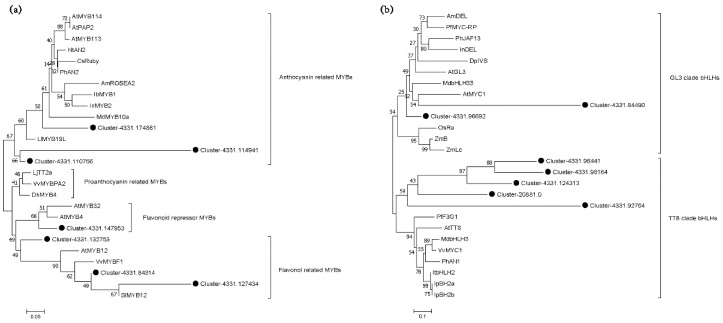
Phylogenetic analysis of (**a**) *LsMYBs* and (**b**) *LsbHLHs* TFs from *L. spicata* and compared with that of other species. Black nodes indicated *LsMYBs* and *LsMYBs*. Functions of all *MYBs* and *bHLHs* were listed on the right. Gene bank accession numbers of the used sequences were listed in Supplementary Table S3.

In addition to structural genes, the accumulation of anthocyanin is also affected by MBW TFs [12]. One hundred and fifty-nine genes encoding MYB, bHLH and WD40 TFs were identified, but only *MYB* and *bHLH* genes were differentially expressed, accounting for nearly 49.1% (78 genes) (Figure 3b). The expression profile of *MYB* and *bHLH* demonstrated four patterns. The first pattern showed a significant uptrend in L2 and significant downtrend in L3, involving 23 *MYB* and 12 *bHLH* genes. Four MYBs (Cluster 4331.127434, 84314, 174861 and 110756) and one bHLH (Cluster 4331.96692) which were clustered in flavonoid-related clades, belonged to the first pattern (Figure 4). Even more importantly, Cluster 4331.96692 was the only DEGs in the *bHLH* gene family. Except for the above genes, the other *MYBs* and *bHLHs* were expressed steadily. The average FPKM of Cluster 4331.110756 (MYB) in L2 was 471.69, 77.95 times higher than that in L1. The second pattern saw a decreased expression level in L2 and an increased expression in L3, involving 18 *MYB* genes and 8 *bHLH* genes. Seventeen genes expressed in the mode of continuous upregulation and downregulation during the three periods, of which 6 genes performed in the former mode and 11 the latter. A total of 78.2% genes expressed in the first two patterns and 21.8% in the last two modes (Supplementary Table S2).

### 2.5. Co-Expression Analysis of Genes Related to the Flavonoid Synthesis Pathway

**Figure 5:**
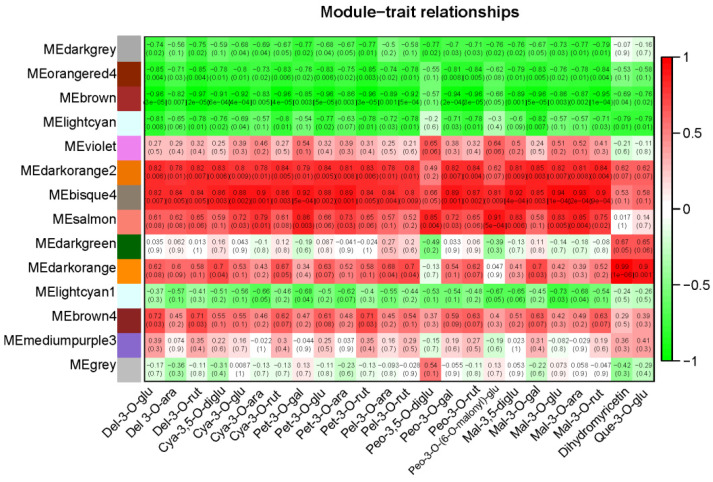
Relationship between module eigengenes (MEs, rows) and traits (columns). MEs were used to create the correlation and its *p* value between modules and traits (24 DAMs). The right panel described a color scale for module–DAMs correlations from –1 to 1.

In order to explore the correlation between genes and distinguish the co-expression genes, 4167 DEGs (mean FPKM > 23) were selected to perform weighted gene co-expression network analysis (WGCNA). In the WGCNA network, the conditions for softpower and cutheight were set as 24 and 0.2, respectively [11,32]. Based on WGCNA program, 4167 DEGs were clustered into 14 major tree branches, with each branch representing a different colored module (Figure S1). The modules were developed to represent the groups of DEGs with high topological overlap and the same expression. The correlation coefficients (r) and *p* values between each module with 24 DAMs were selected as traits, which are shown in Figure 5. When setting the thresholds as *p* < 0.05 and r > 0.5 or r < 0.5 [10], 8 modules were found to be significantly correlated to most of the anthocyanins and flavonols, including 4 positively regulated modules and 4 negative modules (Table 2, Figure 5). Hub genes (highly connected gene), that represent the expression characteristics of each module, were obtained by means of CentiScaPe (http://apps.cytoscape.org/apps/centiscape#cy–app–releases–tab, accessed on 24 October 2021), specifically, by calculating the geometric means of degree centrality, closeness centrality and betweenness centrality. In this study, 38 hub genes were identified and 25 of them had annotations in KEGG database. Eight genes were found to be involved in the synthesis of anthocyanin, with 3 in Bisque4 module and 5 in Darkorange module. The three genes in bisque4 module that regulated anthocyanin synthesis were two *F3′H* (Clust–4331.96380 and Clust–4331.92335) and one *F3′5′H* (Cluster–4331.95769). They expressed 14.26 times, 13.31 times and 16.98 times higher, correspondingly, in L2 than in L1, continuing to upregulate, and by nearly 1.5-fold in L3 over L2. The five genes in the darkorange module included two *UFGTs* (Cluster 4331.91537, Cluster 4331.99009), two *4CLs* (Cluster 4331.72626, Cluster 4331.85224) and one *F3H* (Cluster 4331.81292). Their expressions were significantly upregulated in L2 and downregulated in L3, which were different from the expression of *F3′Hs* and *F3′5′H* in the bisque4 module. Hub genes, located in the center of every module, represented the expression characteristics of each module. In other words, the genes in both the darkorange module and the bisque4 module regulated anthocyanins synthesis, but their expression patterns were slightly different. In addition to the five hub genes, a variety of structural genes and regulatory genes (edge weight > 0.45) involving in anthocyanin synthesis were also clustered in the darkorange module, which included two *CHSs* (Cluster 4331.85043, Cluster 4331.85366), one *4CL* (Cluster 4331.103012), two *C4Hs* (Cluster 4331.47491, Cluster 4331.79255), two *OMTs* (Cluster 4331.93549, Cluster 4331.101486), three *MYBs* (Cluster 4331.174861, Cluster 4331.84314 and Cluster 4331.110756) and one *bHLH* (Cluster 4331.96692). In Cluster 4331.110756, one of the *MYB* genes was expressed as significantly high in level and upregulation in anthocyanin synthesis and, furthermore, was highly corrected with structural genes and regulatory genes including *4CL*, *CHS*, *F3H*, *F3′H*, *OMT*, *bHLH* and *WRKY*. 

In addition, four modules were found to be negatively correlated with the synthesis of anthocyanin. The gene centrality was calculated using the same method, and it was found that most hub genes in the four modules were related to the photosynthesis of *L. spicata* fruit, including photosynthetic carbon fixation proteins, antenna proteins and photosynthetic electron transfer proteins.

### 2.6. Relationship between the Genes Expression and Anthocyanins

**Figure 6:**
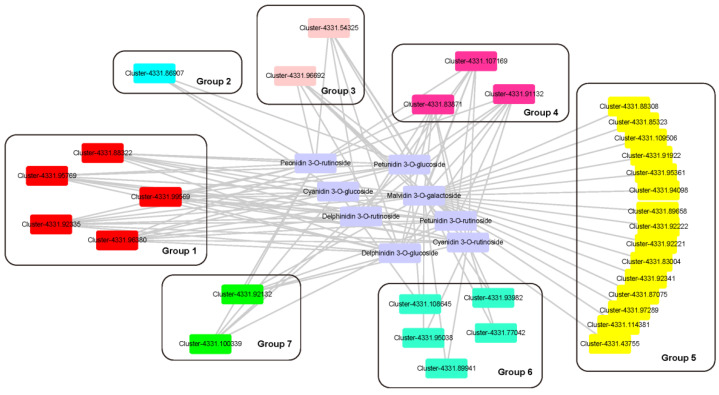
Construction of regulatory networks of anthocyanin biosynthesis. Eight anthocyanins and thirty-six genes were applied to construct the network by means of Cytoscape (3.7.2 version) According to the type of anthocyanins to which genes related, 7 groups were created. The genes located in Group 1 to 7 corresponded to 8, 3, 6, 7, 1, 2, 5 types of anthocyanins, respectively.

To reveal the relationship between anthocyanin accumulation and gene expression in *L. spicata* fruits, the connectivity between eight major anthocyanins and all DEGs involved in anthocyanin synthesis were identified. A total of 33 genes were significantly correlated with major anthocyanins (r > 0.7) (Figure 6). Five genes in Group 1 were significantly associated with eight anthocyanins, of which, two were annotated as *F3′H*, two as *4CL*, and one as *F3′5′H.* The *F3′Hs* (Cluster 4331.92335, and Cluster 4331.96380) and *F3′5′H* (Cluster 4331.95769) were overlapped with the hub genes in WGCNA, which meant that they played an important role in the regulation of anthocyanin synthesis. Three genes clustered in Group 4 were significantly associated with seven major anthocyanins (except Cyanidin 3–*O*–glucoside), which were annotated as *ANS*, *F3′5′H* and *PAL*. The genes located in Group 2, 3, 6, 7 corresponded to 3, 6, 2, 5 types of anthocyanins, respectively. Seven structural genes and three *bHLH* genes were included in the four gene groups, of which, *bHLH* (Cluster 4331.86907) expressed the highest in MBW complex and showed direct connectivity with Cyanidin 3–*O*–glucoside, Peonidin 3–*O*–rutinoside and Petunidin 3–*O*–rutinoside. Group 5 clustered the most genes, but significantly correlated with only one anthocyanin. Except for significant correlation between *CHS* (Cluster–4331.43755) and Cyanidin 3–*O*–rutinoside, the other 14 genes were significantly correlated with Malvidin 3–*O*–galactoside. 

### 2.7. Verification by RT–qPCR

**Figure 7:**
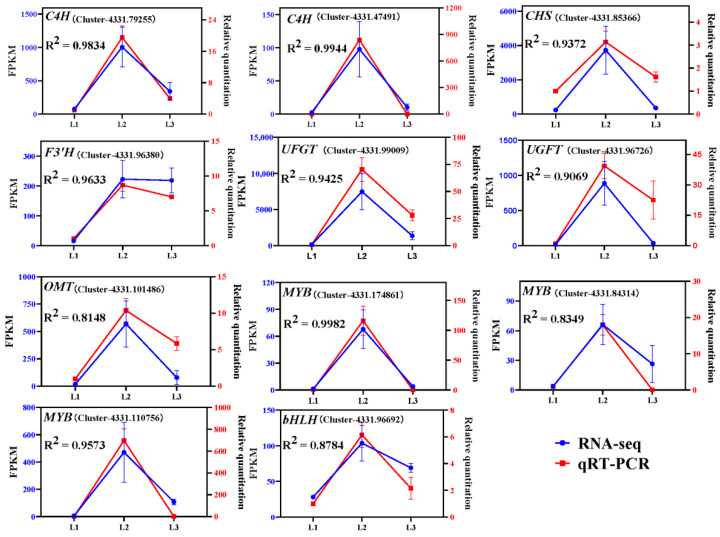
RT–qPCR results of eleven randomly selected hub genes from darkorange modules, bisque4 modules and the anthocyanin–gene correlation network. Error bars represent ± SE of biological replicates (three biological replicates × three stages, *n* = 9).

To verify the accuracy of RNA–seq data, RT–qPCR was conducted on 11 randomly selected DEGs located in the darkorange module and bisque4 module, taking *cation transport mediators* (*CNNM*) and *G protein-coupled receptor 107* (*GPR107*) as reference genes (unpublished). The primers designed are listed in Supplementary Table S4. The results showed that the expression patterns of the genes were almost consistent with the RNA–seq results (R^2^ = 0.8143 – 0.9982) (Figure 7), and accordingly, the RNA–seq data was valid and reliable.

## 3. Discussion

The composition of anthocyanins and their variation in content are the material bases for plant color. Liriope species are characterized as their black fruits, which can be used to distinguish common ground cover such as *L. spicata* and *Ophiopogon japonicas* (blue fruits) [24]. In this study, Cyanidin, Delphinidin and their *O*-methylated products, also named Peonidin, Petunidin and Malvidin (Figure 1d), were found to be the main metabolites in *L. spicata* fruit. However, *O*-methylated products only caused slight redness, and the basic tone was still similar [1,12]. Studies have found that purple/black plant tissues are rich in Delphinidin or Cyanidin. For example, Delphinidin was the dominant type of metabolite in the deep purple tea (*Camellia sinensis*) ‘Ziyan’, but Ultraviolet A/B treatment could increase the total amount of anthocyanin and the proportion of Cyanidin [33]. The black seed-coated bean (*Vigna angularis*) contains large amount of Delphinidin 3–*O*–galactoside and Delphinidin 3–*O*–glucoside [34]. Cyanidin–3–*O*–glucoside was the main anthocyanin in the skin of black rice (*Oryza sativa*) [35]. Cyanidin and Peonidin were the key anthocyanins in *Asparagus officinalis* [36]. In this study, eight main DAMs were identified in *L. spicata* fruit during the color formation period, containing Delphinidin 3–*O*–glucoside, Peonidin 3–*O*–rutinoside, Delphinidin 3–*O*–rutinoside, Petunidin 3–*O*–rutinoside, Malvidin 3–*O*–galactoside, Petunidin 3–*O*–glucoside, Cyanidin 3–*O*–rutinoside and Cyanidin 3–*O*–glucoside. In particular, the content of Peonidin 3–*O*–rutinoside and Delphinidin 3–*O*–glucoside were remarkably high, and the VIP climbed highest after the color transformation. Therefore, the reason for the black skin of *L. spicata* fruit can be explained as the accumulation of the two hydroxylated types of anthocyanins, which dominate the color transformation of fruit. With the increased amount of total anthocyanin, the purple gradually deepens. This color formation pattern was also reported in the bicolor flower of ‘Ratflam’ (Muscari) [3]. In addition to anthocyanins, the colorless flavonols also affected the color formation. The correlation between flavonols and anthocyanins have been reported to have influence on the color of most deep purple flowers and fruits [6,7,19,37]. As the radio of flavonols to anthocyanins in this study was extremely low, substantial accumulation in anthocyanin and the inhibition of flavonols could be considered as the premise for the formation of the black fruit skin of *L. spicata.*

Through multivariate statistical analysis on the metabolome, the key metabolites in black fruits of *L. spicata* were found, but analysis on the final product was inadequate for explaining the mechanism of color formation [38]. Subsequently, the co-expressive regular gene groups were established to reveal more mechanisms of color formation. Based on RNA–seq data and KEGG enrichment analysis, 106 DEGs (17 gene families, Table 1) which involved in the synthesis of anthocyanins and flavonols were firstly obtained, then using WGCNA to establish the gene groups. WGCNA was applied extensively to investigate the mechanism of color development, such as red pear [10], *Lonicera japonica* [30], *Chrysanthemum × morifolium* [39] and *Prunus armeniaca* [40]. In the gene groups that positively related to anthocyanins, eight DEGs were considered as hub genes, and they belonged to five gene families (*4CL, F3H, F3′H, F3′5′H* and *UFGT*). Due to the high connectivity of the hub genes, it was speculated that they played a key role in anthocyanins synthesis. According to the perspective of carbon flow, *4Cl, F3H*, *F3′H* and *F3′5′H* were proposed to catalyze the synthesis of anthocyanins, flavonols and proanthocyanidins [41,42,43,44]; consequently, their high expression laid the foundation for the accumulation of total anthocyanin, which was considered to be the direct reason for the higher TAC in *L. spicata* fruits than that in *O. japonicas* fruits (unpublished). In particular, the expression patterns of *4CL*, *F3′H* and *F3′5′H* were significantly correlated with eight major anthocyanins (R^2^ > 0.7). Similar results were reported in strawberry (*F. × ananassa*) that *4CLs* were significantly correlated with the content of anthocyanin in the red flower petals, and were regulated by R2R3–MYB transcription factor [45]. Comparing the transcriptome results of Morus fruits among different colors (black and white), Huang et al. found that the up–regulated expression of *CHS* and *F3H* led to the large accumulation of an intermediate product named dihydroxanthol in black fruits, which was one of the key factors in color difference [25]. A frame-shifting mutation of the *F3′H* gene in *Euphorbia pulcherrima* cultivar ‘Harvest Orange’ resulted in the premature termination of transcription and the non-functional enzyme was encoded. This induced the anthocyanin synthesis pathway to the Pelargonidin branch and rare orange–red bracts appeared [46]. *F3′5′H* is a key gene for the production of Delphinidin-type gathering in blue flowers of *Senecio cruentus* [47] and *Tulipa gesneriana* [48]. The colored anthocyanins are variable, and will transfer into colorless proanthocyanidins under the action of anthocyanidin reductase (ANR). However, this process can be permanently blocked by glycosylation and, consequently, stable pigment was synthesized [49,50]. In this study, no proanthocyanidins were detected, but anthocyanidins were highly accumulated, which was directly caused by *UFGT* identified as the central gene in *L. spicata*. *UFGT* has also been discovered as the key gene in the synthesis of anthocyanin in grape (*Vitis vinifera*) [49], passion fruit (*Passiflora edulis*) [51], wild pomegranate (*Punica granatum*) [52], etc.

The synthesis of anthocyanin is also controlled by the MBW complex. In this experiment, *WD40* gene expressed steadily during the fruit development, which was similar to the results of purple and white sweet potato (*Ipomoea batatas*) [53]; accordingly, WD40 was presumedly not the key TF for the color transformation of *L. spicata*. It is well known that the binary complex formed from MYB TF and bHLH TF regulate the accumulation of flavonoids. Wang et al. reported that the expression of the *SsMYB1* gene determined the leaf color of *Sapium sebiferum*, but in need of the participation of *SsbHLH1* gene. When the *SsMYB1* gene combined with the *SsbHLH1* gene, the promoter of *SsDFR* and *SsANS* would be effectively activated, and the pigmentation of transformed tobacco (*Nicotiana tabacum*) enhanced [54]. In *Centaurea Cyanus*, the highest expression levels of *CcMYB6–1* and *CcbHLH1* were found in Black flower cultivar ‘Tall Double Ball Black’, and the interaction between *CCMYB6–1* and *CCbHLH1* enhanced the transcriptional activities of *CcF3H* and *CcDFR* [17]. A similar binary complex for anthocyanin regulation was reported as MiMYB1/MibHLH218 in red mango (*Mangifera indica*) [18] and PdMYB118/PdTT8 in (*P. davidiana × P. bolleana*) [55]. These results proved that the synergistic effect between MYB and bHLH was most important for anthocyanin synthesis, and the activation of MYB was strongly dependent on bHLH. In the study of *L. spicata*, *MYB* gene (Cluster 4331.110756) had direct connectivity with both structural genes (*4CL, CHS, F3H, F3′H* and *OMT*) and TFs genes (*bHLH* and *WRKY*). The *bHLH* (Cluster 4331.86907) gene was co-expressed with three kinds of anthocyanins including Cyanidin 3–*O*–glucoside, Peonidin 3–*O*–rutinoside and Petunidin 3–*O*–rutinoside. Both *MYB* and *bHLH* were the highly expressed TFs genes in L2. Based on the dual-level omics analysis, we concluded that *L. spicata* had a binary regulating complex for anthocyanin synthesis, but more studies are required to verify this.

## 4. Materials and Methods

### 4.1. Plant Materials

*L. spicata* is widely cultivated in China and can be collected without restriction. Their fruits were allowed to collect from the campus of Zhejiang A&F University, Hangzhou, China (N 29°56′ to 30°23′, E 118°51′ to 119°52′). The randomly scattered fifteen to twenty plants of the similar area were selected as sampling plants. Three growth stages of the fruits based on their color and size were collected, and the collection dates were September, November and December of the year 2020, corresponding to the first stage (L1), the second stage (L2), and the third stage (L3), respectively. The freshly obtained fruits were placed in liquid nitrogen for 30 min and stored in a freezer (−80 °C) for use.

### 4.2. Determination on the Pigment Content 

Then, 200 mg fruits were ground into powder in 200 µL absolute ethyl alcohol (EA). After that, they were sealed and kept in a dark place until the chlorophyll in the crushed fruits was dissolved completely in supernatant liquid. The extracts were collected after filtrating the crushed fruits, followed by determination under spectrophotometer at the absorbances of 470 nm, 649 nm, and 665 nm, respectively. The TCC was determined by means of spectrophotometry with the concentration of chlorophyll below 0.68 cm^2^·mL^−1^ [56]. A solvent composed of EA, distilled water (H2O) and hydrogen chloride (HCl) was prepared at the volume ratio of 80:20:0.3 and was used to extract anthocyanin at 4 °C in the dark condition [57]. The extracts were then detected at the absorbances of 530 nm and 600 nm. The TAC was obtained referring to Chu’s method [58].

### 4.3. Preparation for Metabonomic Samples

The fruits, after freeze–drying, were crushed and ground into a powder at 30 Hz for 1.5 min using a mill (MM 400, Retsch Co., Arzberg, Germany). Then, 50 mg powdered fruits were extracted by 0.5 mL mixed solution formulated with EA, H2O and HCl (799:200:1, *V*/*V*/*V*), firstly by vortex extraction for 10 min, and then ultrasonic extraction for another 10 min. The extraction procedure was repeated twice. After centrifuging at 12,000× *g* rpm under 4 °C for 3 min, the extractions were gathered and filtrated (PTFE, 0.22 μm; Anpel) to conduct LC–MS/MS analysis.

### 4.4. UPLC Conditions

UPLC–ESI–MS/MS system equipped with the WatersACQUITY (Waters, Milford, MA, USA) (1.7 μm, 2.1 mm × 100 mm) column was applied to analyze the extracts (UPLC, Shim–pack UFLC SHIMADZU CBM30A system, Shimadzu; MS, Applied Biosystems 6500 Triple Quad–rupole, Sciex, ON, Canada). The mobile phase consisted of water and methanol with 0.1% formic acid in both solutions, and the elution was carried out following the gradient program with an injection volume of 2 μL, initially eluted in a volume ratio of 95:5, followed by 50:50 at 6 min, then 5:95 at 12 min for 2 min, finally 95:5 at 14 min, holding for 2 min. The flow rate of the moving phase was fixed at 0.35 mL/min and the column temperature was maintained at 40 °C. The detection and quantification of effluent were performed on ESI-triple quadrupole-linear ion trap (QTRAP)–MS (San Diego, CA, USA) and the operating parameters were provided in Table S5. The metabolites of anthocyanidin eluted within the period were quantified by Multiple Reaction Monitoring (MRM). Flavonoid contents were detected by MetWare (http://www.metware.cn/ accessed on 4 November 2021, Wuhan, China) based on HPLC and the ESI–Q TRAPMS/MS platform [59]. The detailed information of standard analysis and the chromatograms of the flavonoid compounds are provided in Table S6 and Figure S2, respectively.

### 4.5. Total RNA Extraction and Quality Analysis

RNA extraction kit (TIANGEN RNAprep FFPE Kit, Beijing, China) was used to extract the total RNA by following the manufacturer’s protocols. The purity and concentration of the obtained RNA were examined by a NanoDrop™ One/OneC system (Thermo Fisher Scientific, Waltham, MA, USA). The integrity of RNA was assessed using Agilent 2100 Bioanalyzer (Agilent Technologies Inc., Santa Clara, CA, USA) and agarose gel electrophoresis.

### 4.6. Illumina RNA–Seq Library Construction, Expression Level Estimation and Phylogenetic Analysis

Fruits from three stages were collected for RNA sequencing, with three biological replicates for each stage; thus, nine sequencing libraries were generated. A total amount of 1 µg RNA per sample was used to establish the library by means of NEBNext^®^ UltraTM RNA Library Prep Kit for Illumina^®^ (Illumina Inc., San Diego, CA, USA). The quantitation and quality of library was examined using Qubit2.0 Fluorometer and Agilent 2100 bioanalyzer (Agilent Technologies, Santa Clara, CA, USA), correspondingly. Subsequently, the libraries were sequenced on an Illumina Novaseq platform. A number of 150 bp paired-end reads were finally generated after filtering the low-quality reads. 

The expression level of genes was evaluated according to the fragments per kilobase of transcript-per-million-mapped reads (FPKM), which was computed using featureCounts (version 1.5.0). DEGs between the stages were identified by the DESeq2 R package (version 1.16.1). To minimize the false discovery rate, the final *p* values were calibrated following the Benjamini–Hochberg method. Genes with a corrected value of *p* < 0.05 were considered as DEGs (fold change ≥ 2, false-discovery rate <0.01).

The phylogenetics of *MYBs* and *bHLHs* were created by means of MEGA 7.0 with the Neighbor Joining method. The conserved domain of sequences was gained by Gblocks, (http://molevol.cmima.csic.es/castresana/Gblocks_server.html accessed on 4 November 2021). Bootstrap values are for 1000 replicates.

### 4.7. QRT–PCR Analysis

An amount of 1 mg total RNA was synthesized into the cDNA using PrimerScript™ RT Master Mix cDNA kit (TaKaRa, Tokyo, Japan). The primer sequences for each isoform/unigene were designed by primer 5. The obtained cDNA samples were analyzed by qRT–PCR in a 20 μL reacting solution formulated with TB Green™ Premix Ex Taq™ (TaKaRa, Tokyo, Japan), primer pairs and double-distilled water. The qRT–PCR procedure was performed as follows: initial denaturation at 95 °C for 30 s, followed by 40 cycles of denaturation for 5 s at 95 °C and annealing for 15 s at 60 °C. The qRT–PCR analysis was performed on a Light-Cycler 480II real-time PCR detection system (LightCycler^®^ 480 II Roche cycler, Roche, Carlsbad, CA, USA).

### 4.8. Statistical Analysis

The variable importance in projection (VIP) was carried out using SIMCA 14. One-way ANOVA was performed by means of Duncan’s multiple range test of the Statistical Product and Service Solutions program (version 19) (SPSS Inc., Chicago, IL, USA); then, the graphs were plotted by Graphpad prism 8.0 (GraphPad Software, San Diego, CA, USA).

## 5. Conclusions

This study expounded on the material basis and molecular regulation mechanisms for the color formation of *L. spicata* fruit. During the fruit development, 24 DAMs were screened, of which Peonidin 3–*O*–rutinoside and Delphinidin 3–*O*–glucoside accounts played an important role in the color formation of *L. spicata* fruit. Moreover, a series of hub genes including *4CL*, *F3H*, *F3′H, F3′5′H* and *UFGT* were upregulated, and they were corrected with regulatory genes including *MYB* and *bHLH*. This result provides candidate genes for further study, in order to identify the mechanisms of anthocyanin accumulation in *L. spicata* fruit.

## Figures and Tables

**Table 1:** Candidate unigenes involved in anthocyanin biosynthesis, flavonol biosynthesis in *L. spicata* fruit skin. Ⅰ: Shared biosynthesis genes of anthocyanin and flavonol. Ⅱ: Specific biosynthesis genes of anthocyanin. Ⅲ: Specific biosynthesis genes of flavonol. Ⅳ: Three common transcription factors that regulate flavonoid synthesis. DEGs were selected from each adjoining stage. (L1 to L2, L2 to L3).

	Genes	Description	DEGs	ALL
Ⅰ	*PAL*	phenylalanine ammonia–lyase	15	20
	*C4H*	trans–cinnamate 4–monooxygenase	9	10
	*4CL*	4–coumarate–CoA ligase	22	70
	*CHS*	chalcone synthase	20	22
	*CHI*	chalcone isomerase	8	24
	*F3H*	naringenin 3–dioxygenase	6	6
	*F3′5′H*	flavonoid 3′,5′–hydroxylase	2	2
Ⅱ	*DFR*	dihydroflavonol 4–reductase	0	2
	*LDOX/ANS*	leucoanthocyanidin dioxygenase	6	6
	*UFGT*	anthocyanidin 3–*O*–glucosyltransferase	3	5
	*OMT*	caffeoyl–CoA *O*–methyltransferase	7	14
Ⅲ	*FLS*	flavonol synthase	3	7
	*FG3*	flavonol–3–*O*–glucoside	2	2
Ⅳ	*MYB*	transcription factor MYB	52	88
	*bHLH*	transcription factor bHLH	26	57
	*WD40*	transcription factor WD40	0	14

**Table 2:** Statistics of module traits with high correlations.

Group	Module	Response	DEGs
Ⅰ	darkorange2, bisque4, salmon, darkorange	Positively respond to DAMs	1609
Ⅱ	Darkgrey, orangered4, brown, lightcyan	Negatively respond to DAMs	1698

## Data Availability

The data presented in this study are available on request from the corresponding author. The data are not publicly available due to the further study required.

## Supplementary Materials

The following supporting information can be downloaded at: https://www.mdpi.com/article/10.3390/metabo12020144/s1, Figure S1: Dendrogram of 4167 DEGs obtained via hierarchical clustering of topological overlapping dissimilarity. Figure S2: The chromatograms of flavonoids. Table S1: The content of metabolites in *L. spicata* fruits during three stages. Data from three biological replicates are shown, Table S2: Differentially expressed genes involved in flavonoid biosynthesis in *L. spicata* fruits, Table S3: Gene bank accession numbers of the sequences, Table S4: The primer sequences of genes used for quantitative real time PCR. Table S5: The operating parameters of the mass spectrometer, Table S6: The detailed information of standard analysis.
