# Supplementary material for: Integrative Analysis of Metabolome and Transcriptome Reveals the Mechanism of Color Formation in Liriope spicata Fruit
Source: Metabolites. 2022 Feb 4;12(2):144. doi: 10.3390/metabo12020144 (PMC8879266; doi:10.3390/metabo12020144)
Supplement: Supplementary file 1 [file metabolites-12-00144-s001.zip › Figure S1.pdf]

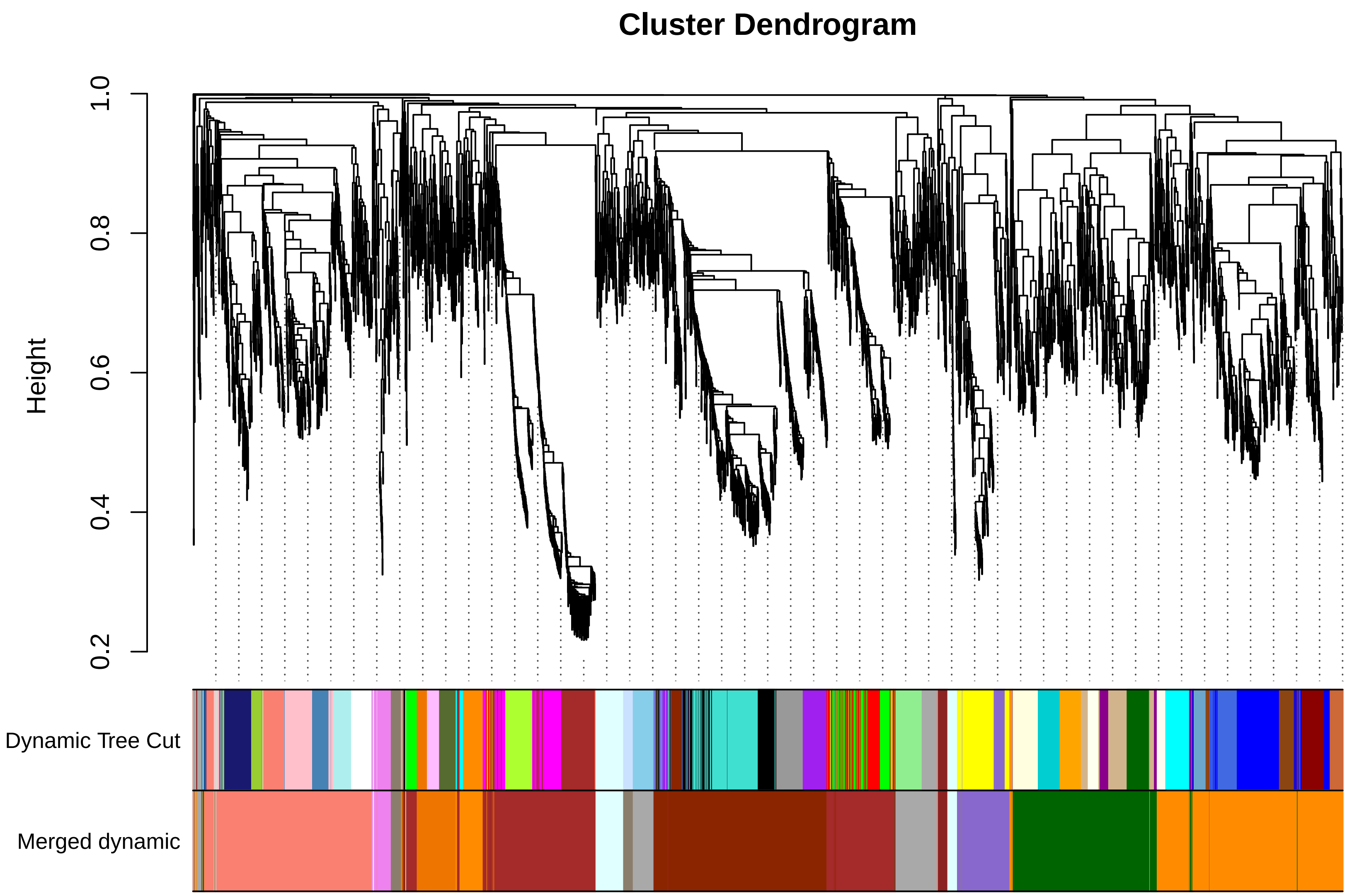

Figure S1. Dendrogram of 4167 DEGs obtained via hierarchical clustering of topological overlapping dissimilarity.
