# Supplementary material for: Integrative Analysis of Metabolome and Transcriptome Reveals the Mechanism of Color Formation in Liriope spicata Fruit
Source: Metabolites. 2022 Feb 4;12(2):144. doi: 10.3390/metabo12020144 (PMC8879266; doi:10.3390/metabo12020144)
Supplement: Supplementary file 1 [file metabolites-12-00144-s001.zip › Figure S2.pdf]

Acq. File:
Anthocy_WH6500-3_BEH-XL-1_V2.0_MHX_EPI_20210511.wiff

Sample Name: STD_5000ppb_5000_CE35
Sample Number: N/A

Cyanidin-3,5-O-diglucoside

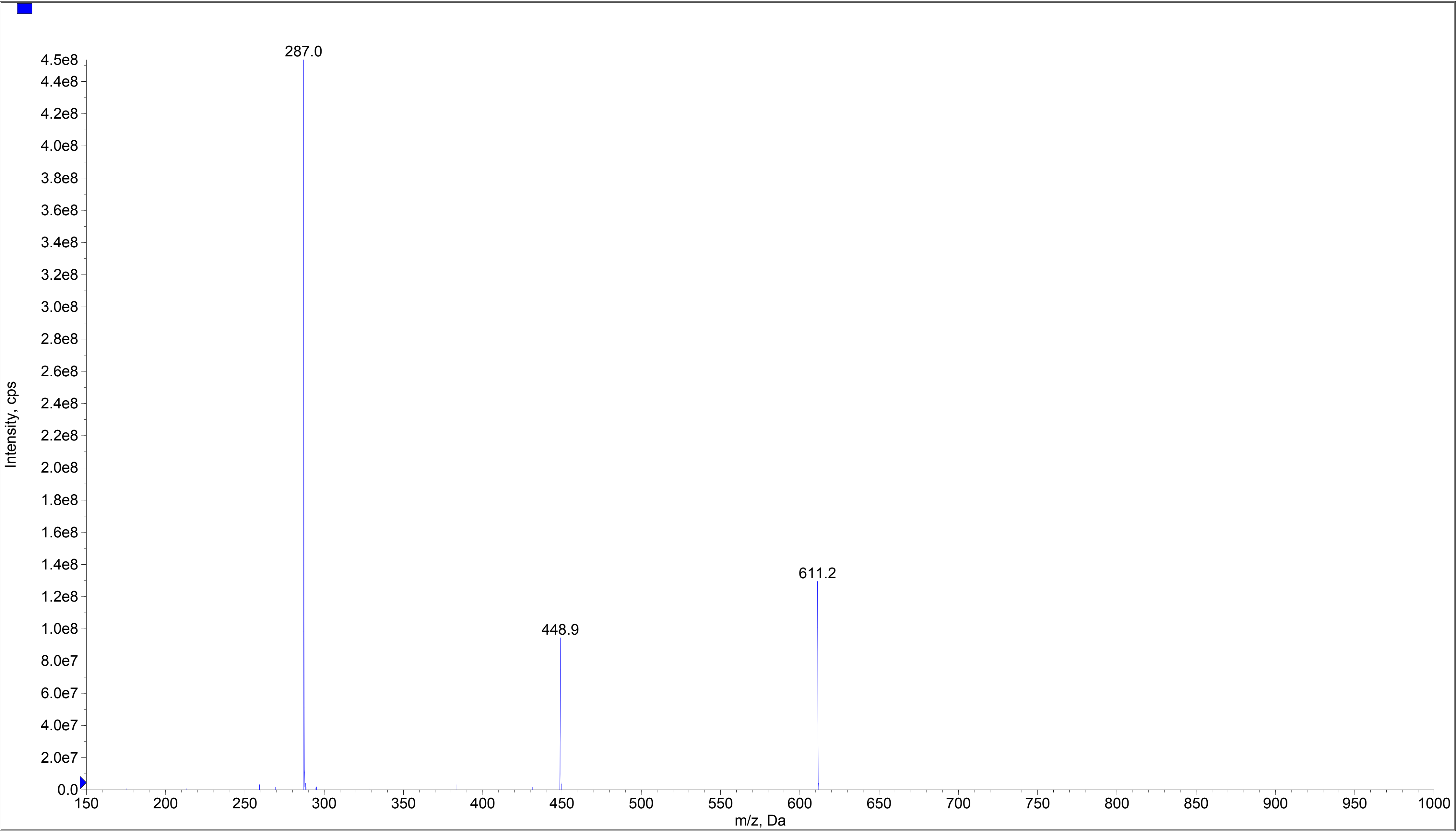

Acq. File:
Anthocy_WH6500-3_BEH-XL-1_V2.0_MHX_EPI_20210511.wiff

Sample Name: STD_2000ppb_5000_CE35
Sample Number: N/A

Cyanidin-3-O-(6-O-malonyl-beta-D-glucoside)

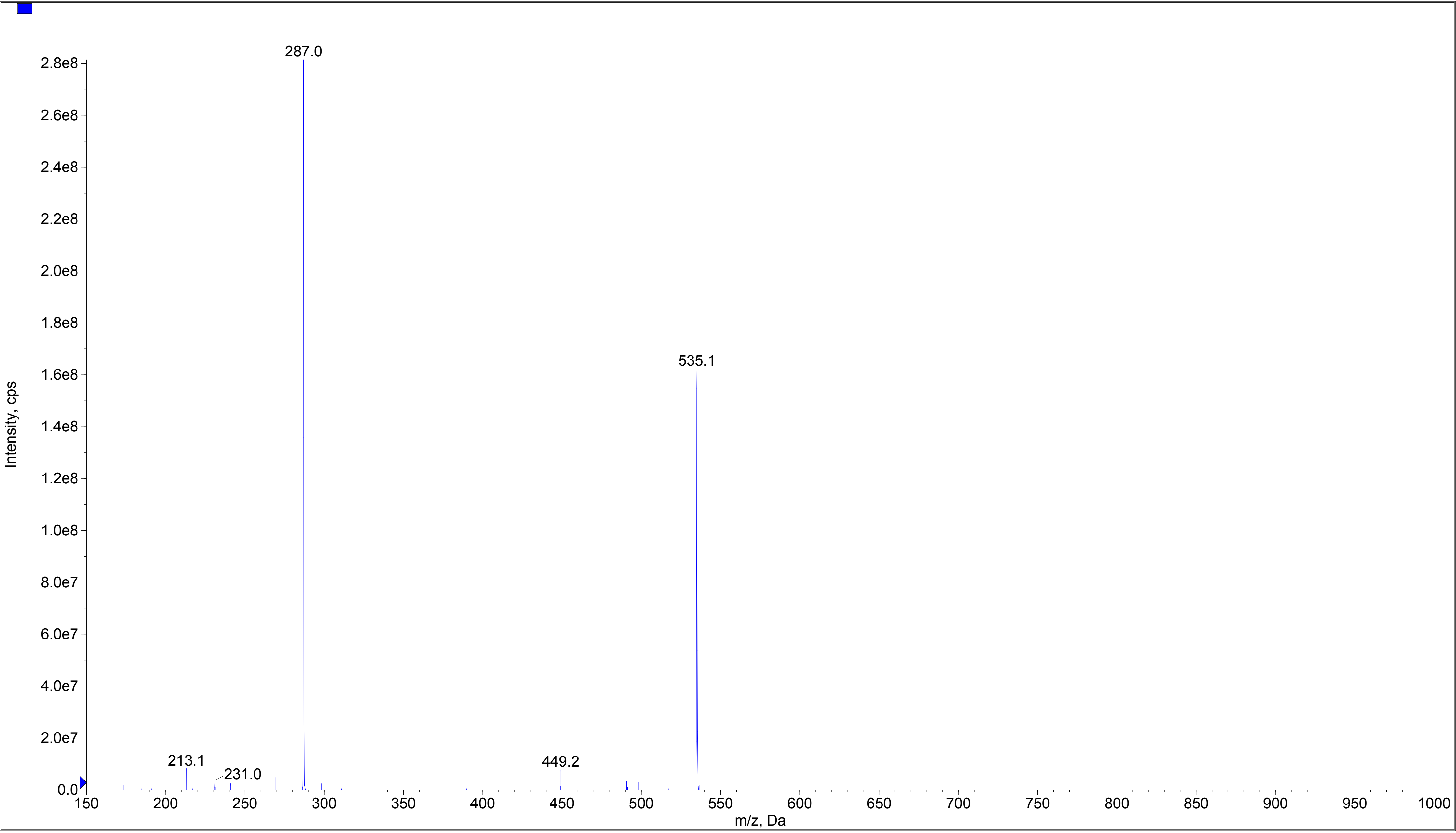

Acq. File:
Anthocy_WH6500-3_BEH-XL-1_V2.0_MHX_EPI_20210511.wiff

Sample Name: STD_2000ppb_5000_CE35
Sample Number: N/A

Cyanidin-3-O-arabinoside

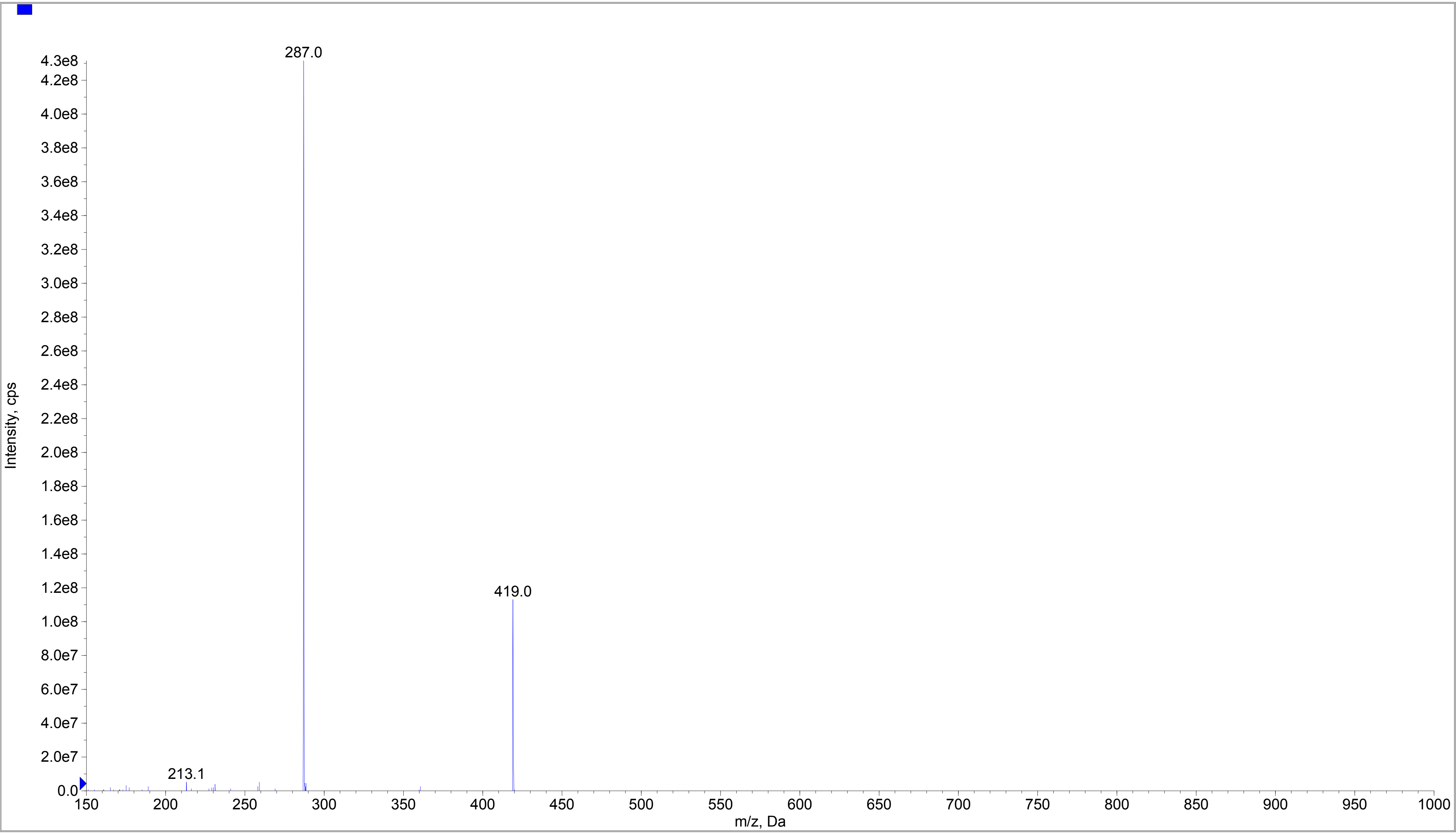

Acq. File: Anthocy_WH6500-3_BEH-XL-1_V2.0_MHX_EPI_20210511.wiff

Sample Name: STD_2000ppb_5000_CE35
Sample Number: N/A

Cyanidin-3-O-glucoside

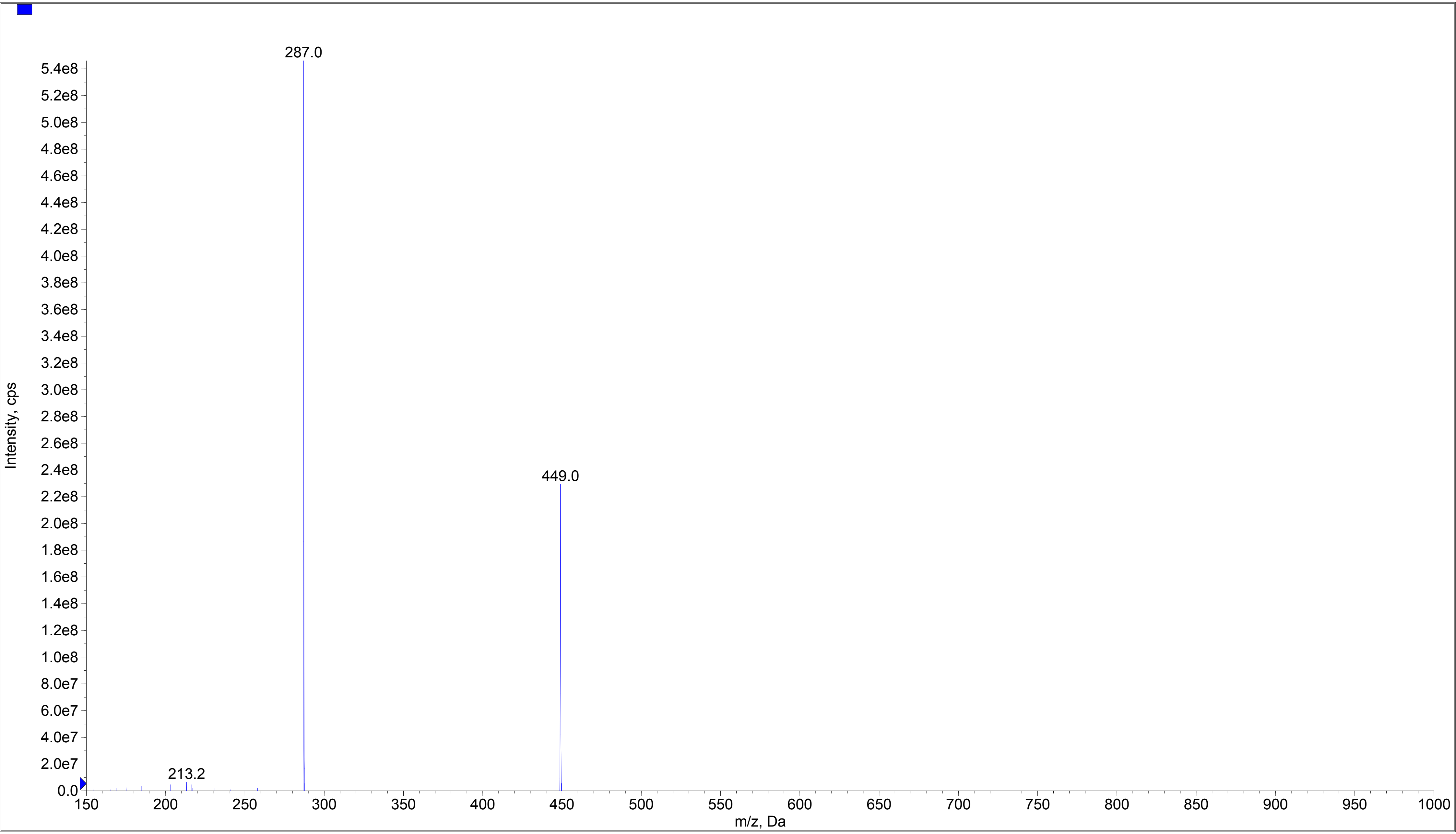

Acq. File:
Anthocy_WH6500-3_BEH-XL-1_V2.0_MHX_EPI_20210511.wiff

Sample Name: Anthocy_mix sample_5000_CE40
Sample Number: N/A

Cyanidin-3-O-rutinoside

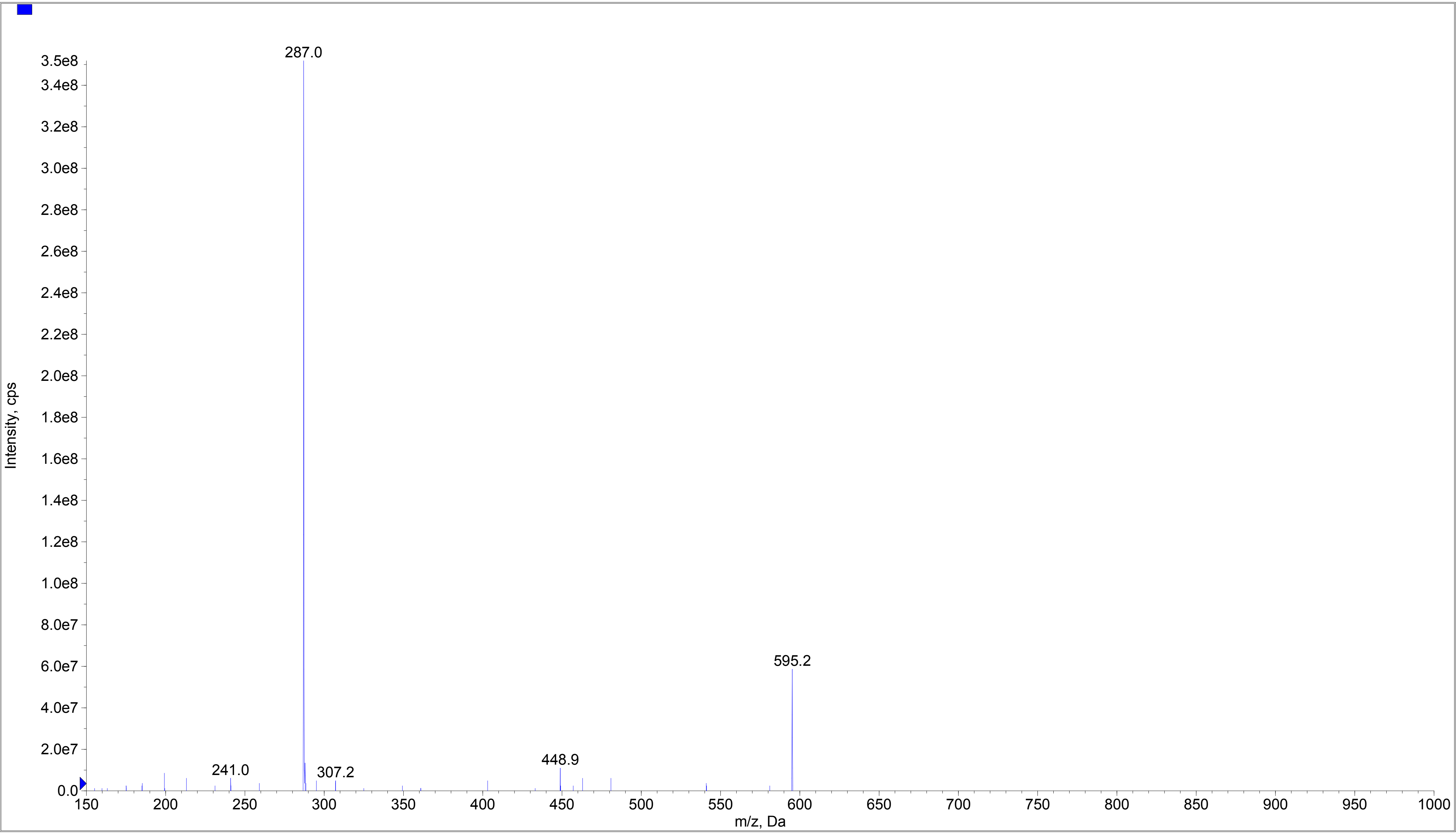

Acq. File:
Anthocy_WH6500-3_BEH-XL-1_V2.0_MHX_EPI_20210511.wiff

Sample Name: A21023226a_a_5000_CE40
Sample Number: N/A

Delphinidin-3-O-(6-O-malonyl-beta-D-glucoside)

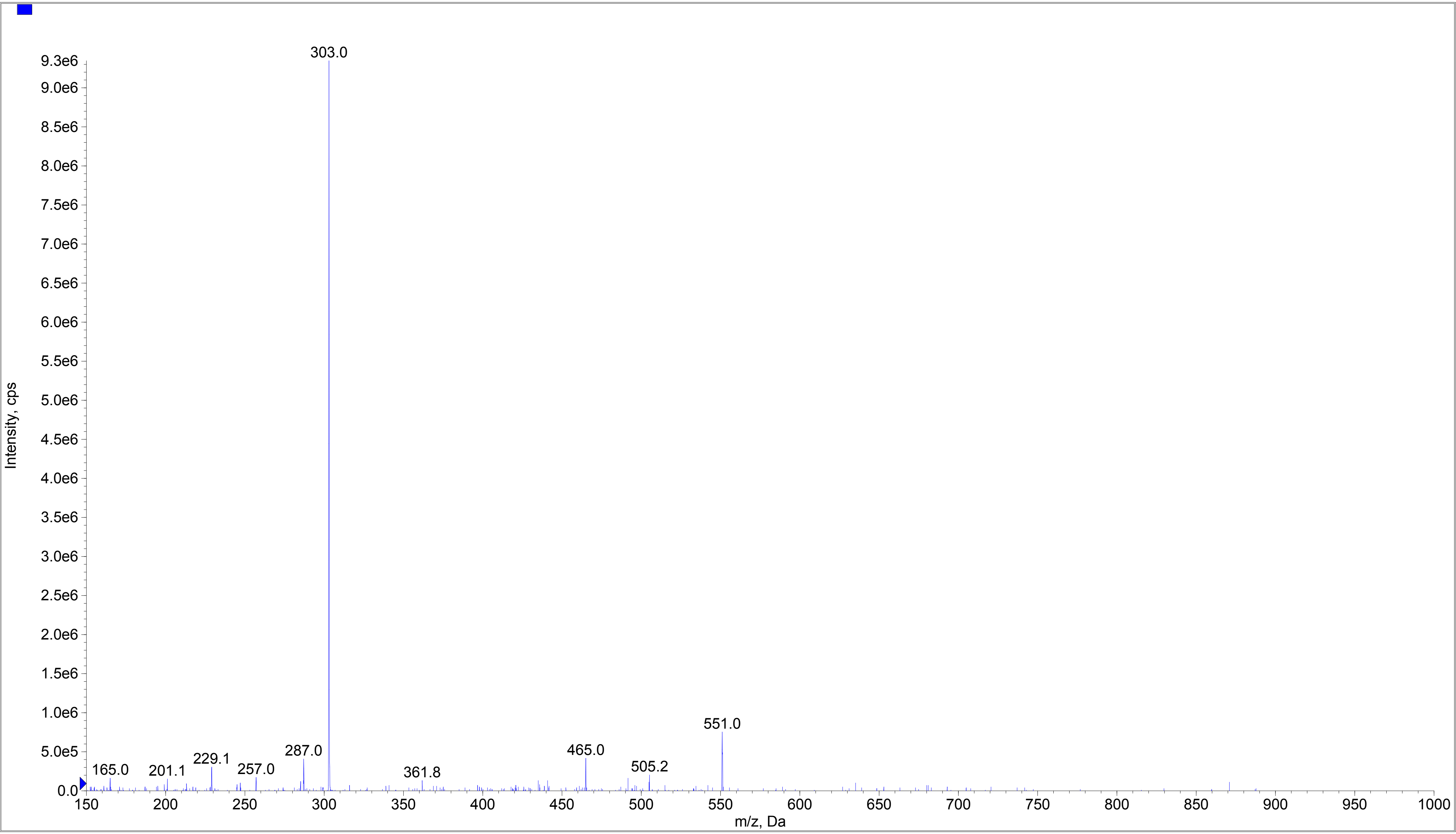

Acq. File:
Anthocy_WH6500-3_BEH-XL-1_V2.0_MHX_EPI_20210511.wiff

Sample Name: Anthocy_mix sample_5000_CE40
Sample Number: N/A

Delphinidin-3-O-arabinoside

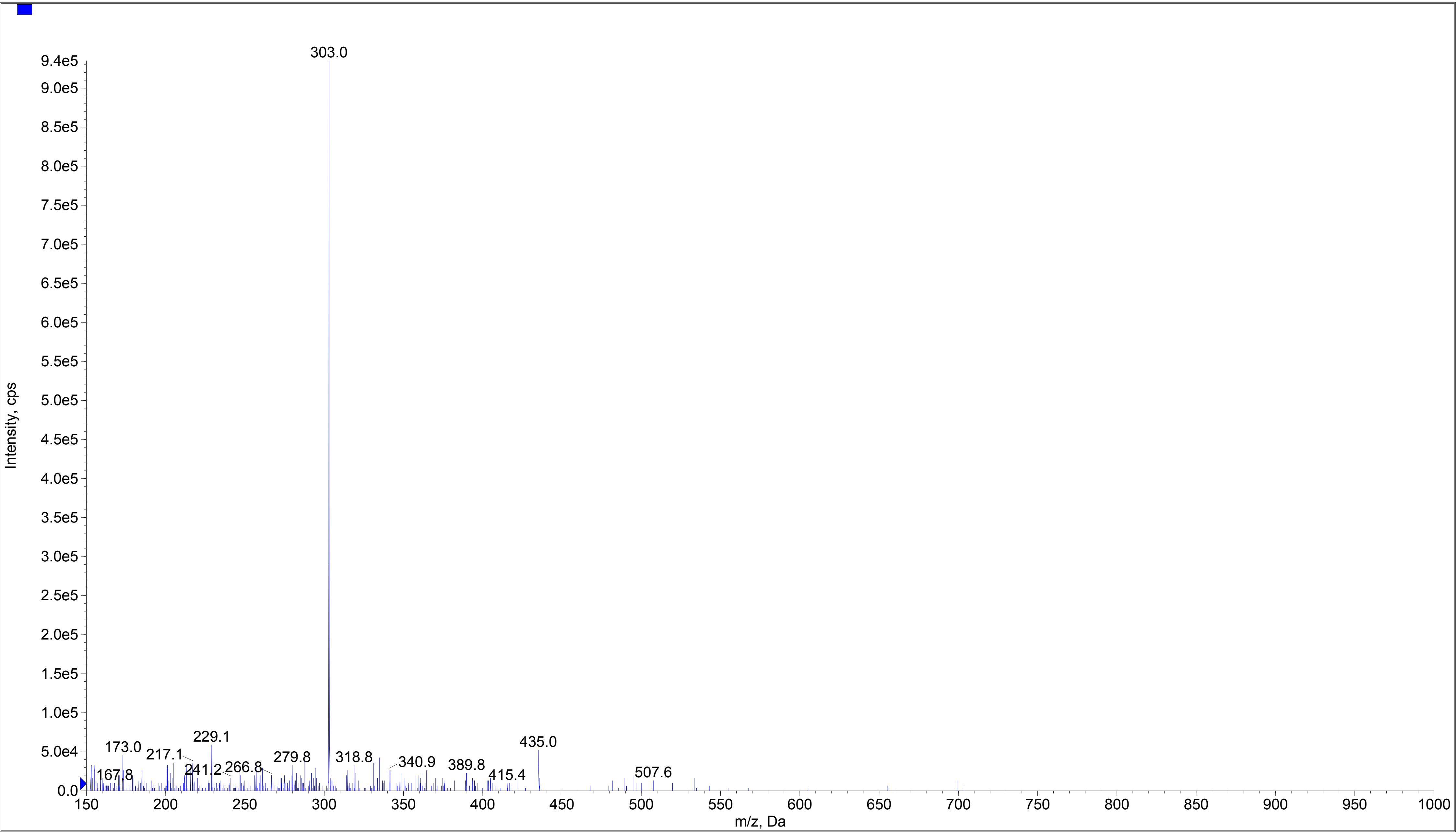

Acq. File:
Anthocy_WH6500-3_BEH-XL-1_V2.0_MHX_EPI_20210511.wiff

Sample Name: STD_5000ppb_5000_CE35
Sample Number: N/A

Delphinidin-3-O-galactoside

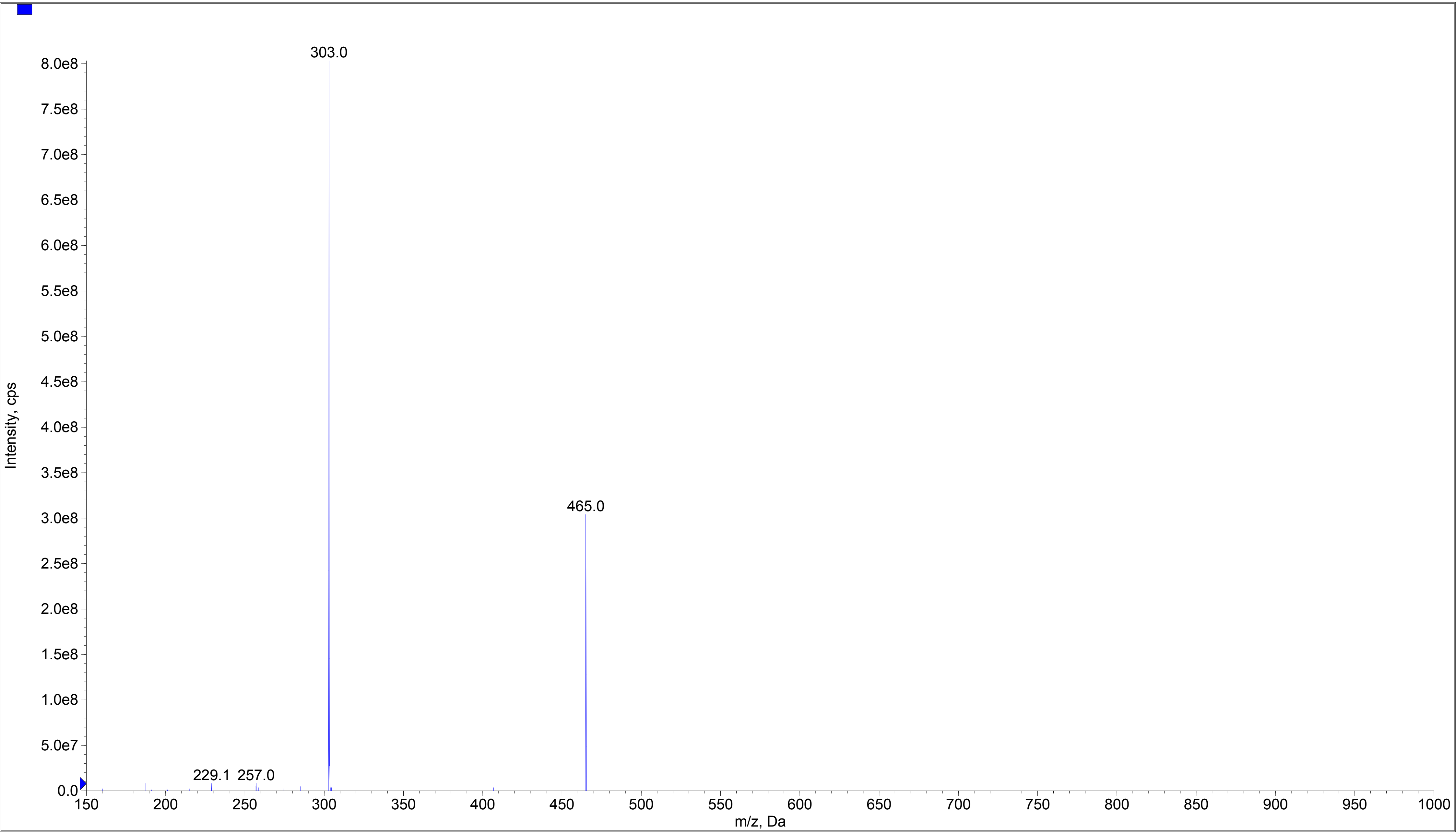

Acq. File: Anthocy_WH6500-3_BEH-XL-1_V2.0_MHX_EPI_20210511.wiff

Sample Name: STD_5000ppb_5000_CE35
Sample Number: N/A

Delphinidin-3-O-glucoside

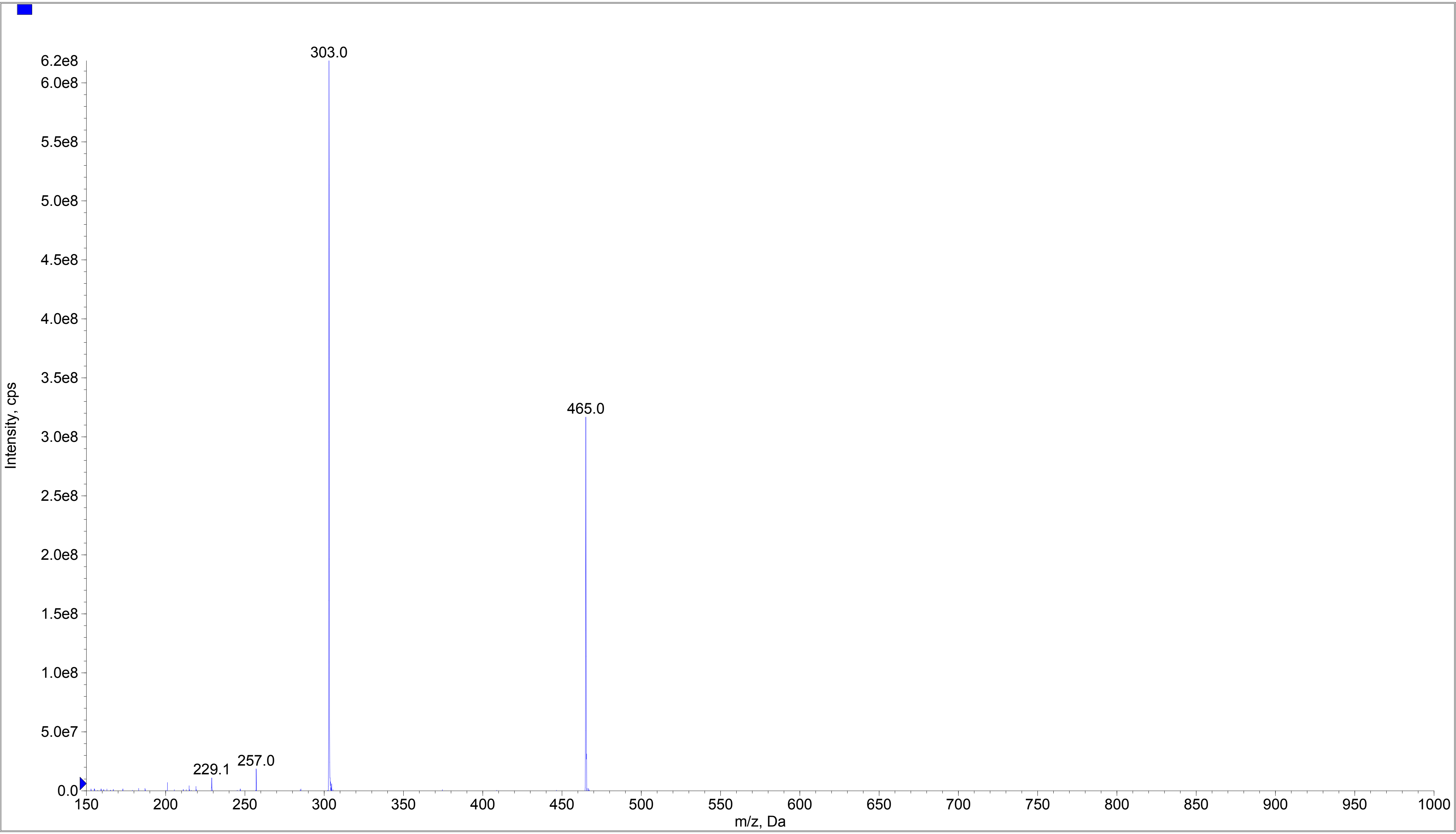

Acq. File:
Anthocy_WH6500-3_BEH-XL-1_V2.0_MHX_EPI_20210511.wiff

Sample Name: STD_5000ppb_5000_CE40
Sample Number: N/A

Delphinidin-3-O-rutinoside

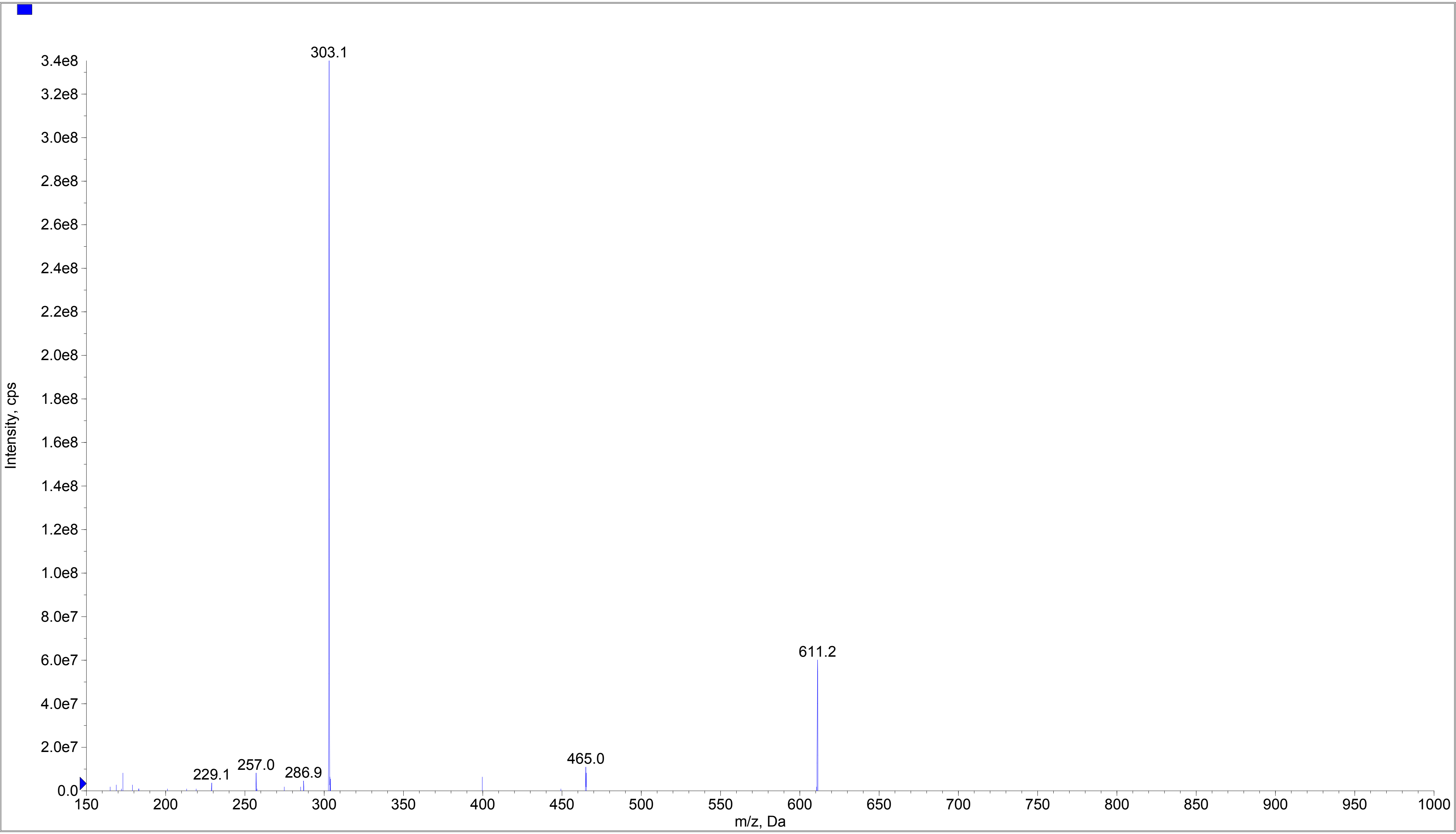

Acq. File: Anthocy_WH6500-3_BEH-XL-1_V2.0_MHX_EPI_20210511.wiff

Sample Name: STD_5000ppb_5000_CE35
Sample Number: N/A

Dihydromyricetin

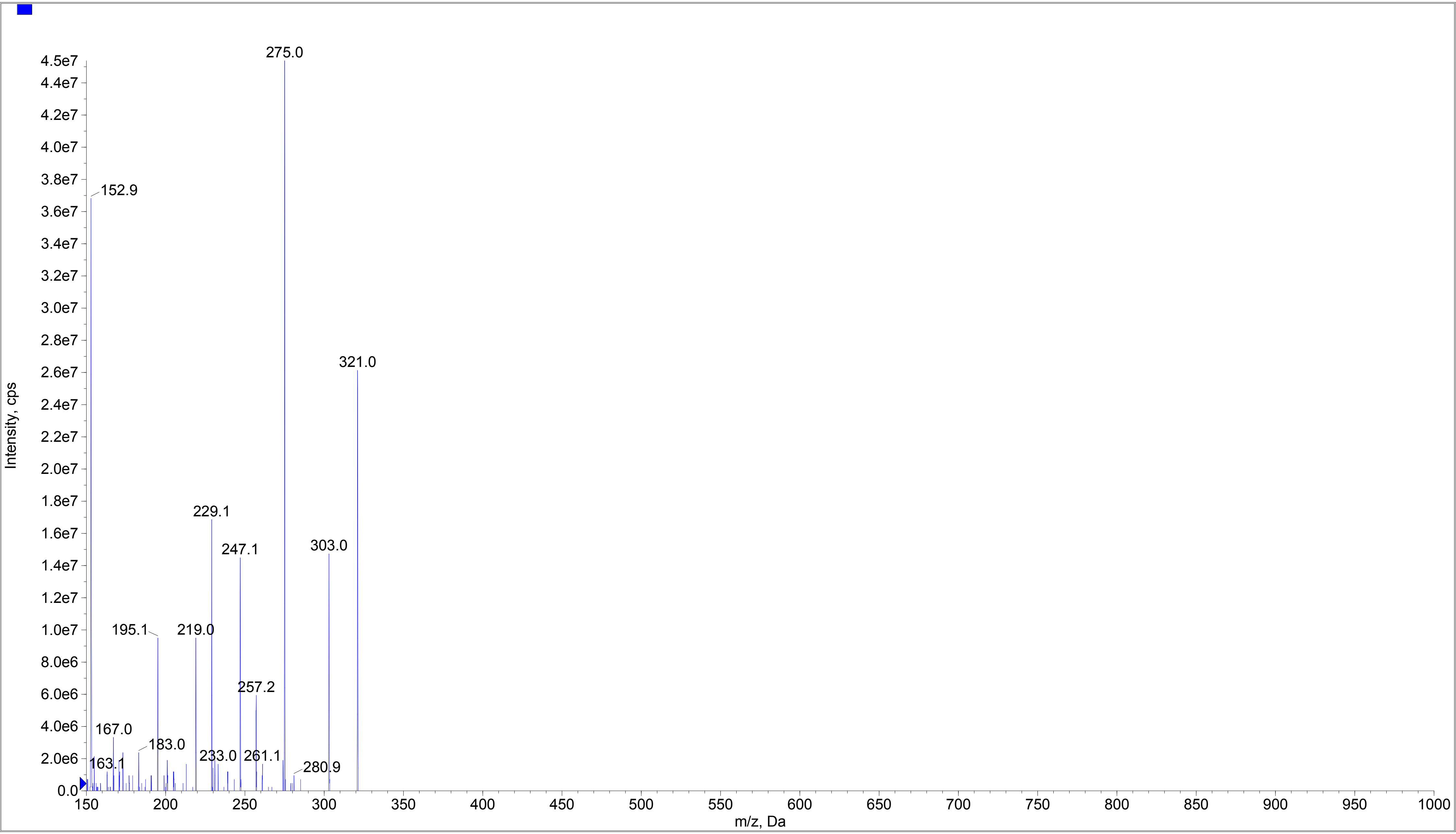

Acq. File: Anthocy_WH6500-3_BEH-XL-1_V2.0_MHX_EPI_20210511.wiff

Sample Name: STD_5000ppb_5000_CE35
Sample Number: N/A

Malvidin-3,5-O-diglucoside

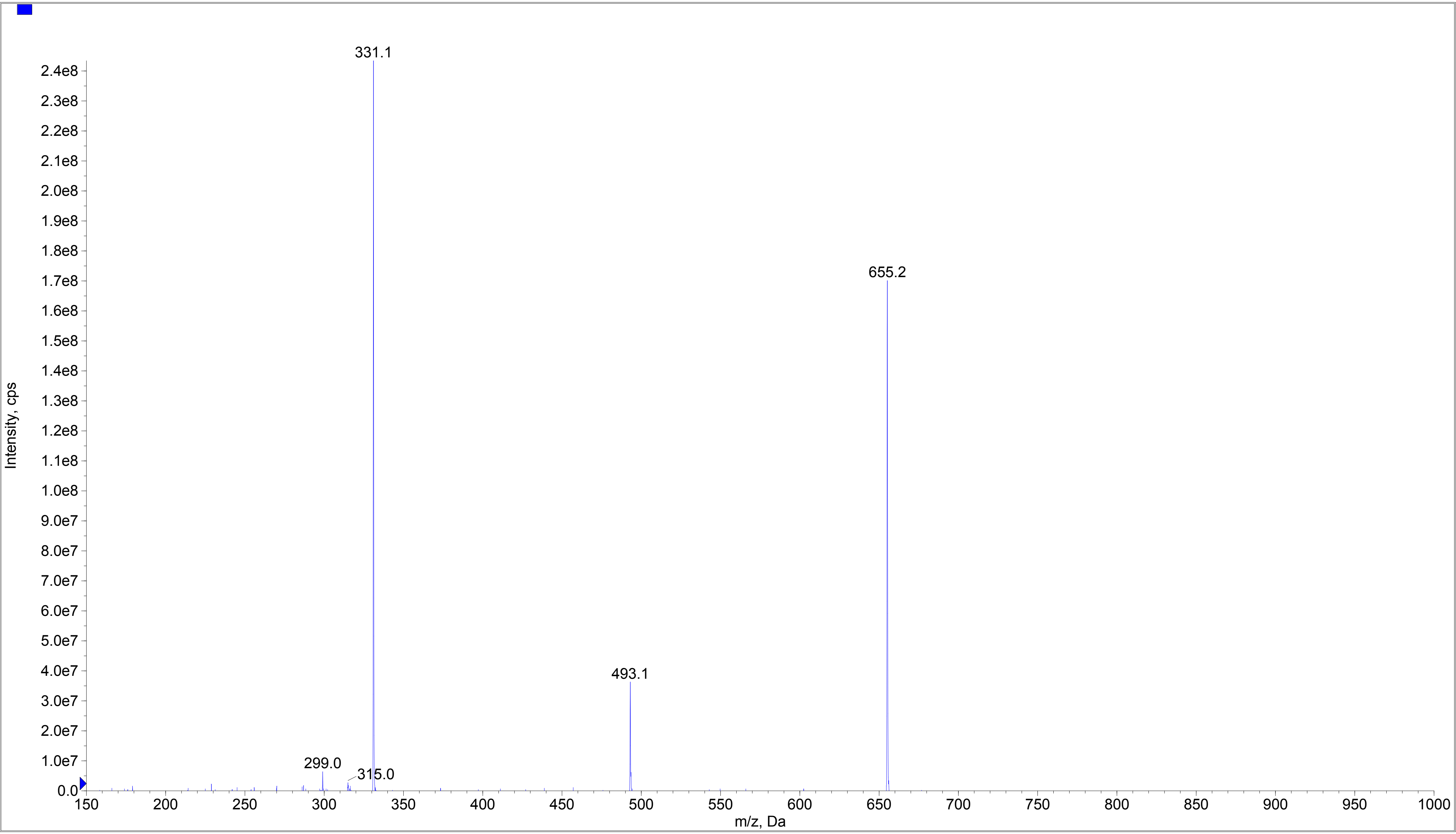

Acq. File:
Anthocy_WH6500-3_BEH-XL-1_V2.0_MHX_EPI_20210511.wiff

Sample Name: STD_1000ppb_5000_CE35
Sample Number: N/A

Malvidin-3-O-arabinoside

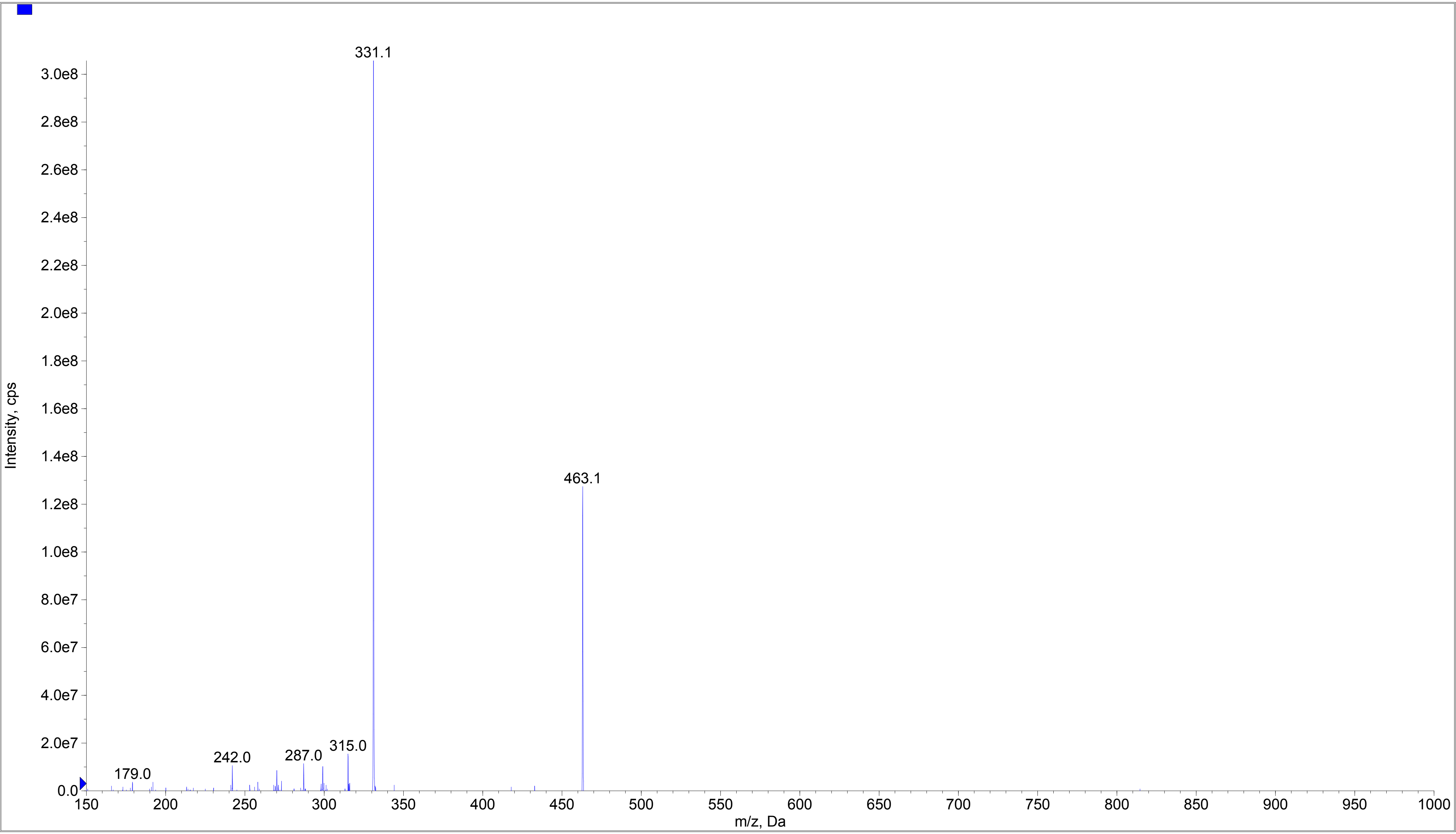

Acq. File: Anthocy_WH6500-3_BEH-XL-1_V2.0_MHX_EPI_20210511.wiff

Sample Name: STD_2000ppb_5000_CE35
Sample Number: N/A

Malvidin-3-O-galactoside

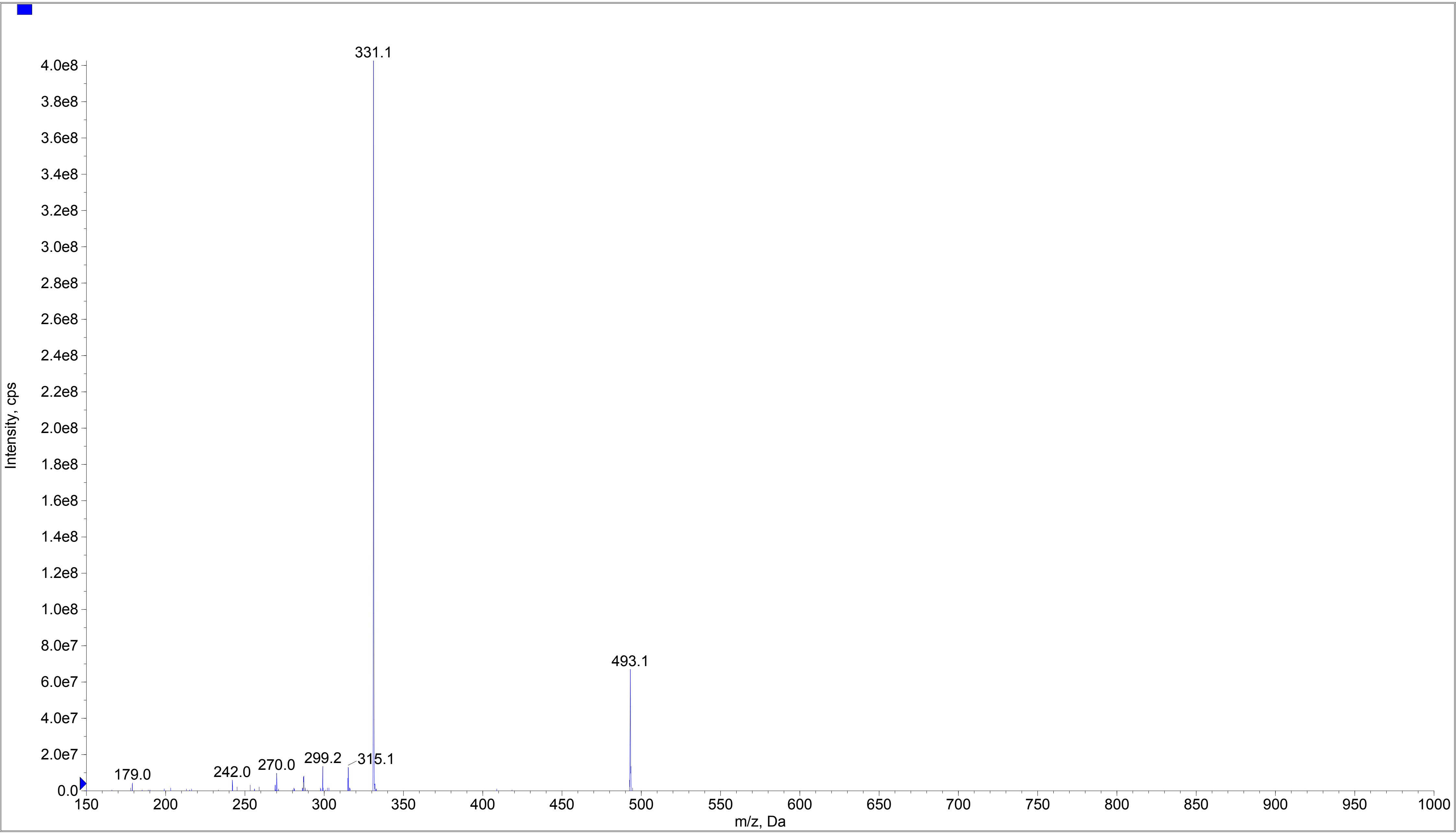

Acq. File: Anthocy_WH6500-3_BEH-XL-1_V2.0_MHX_EPI_20210511.wiff

Sample Name: STD_1000ppb_5000_CE35
Sample Number: N/A

Malvidin-3-O-glucoside

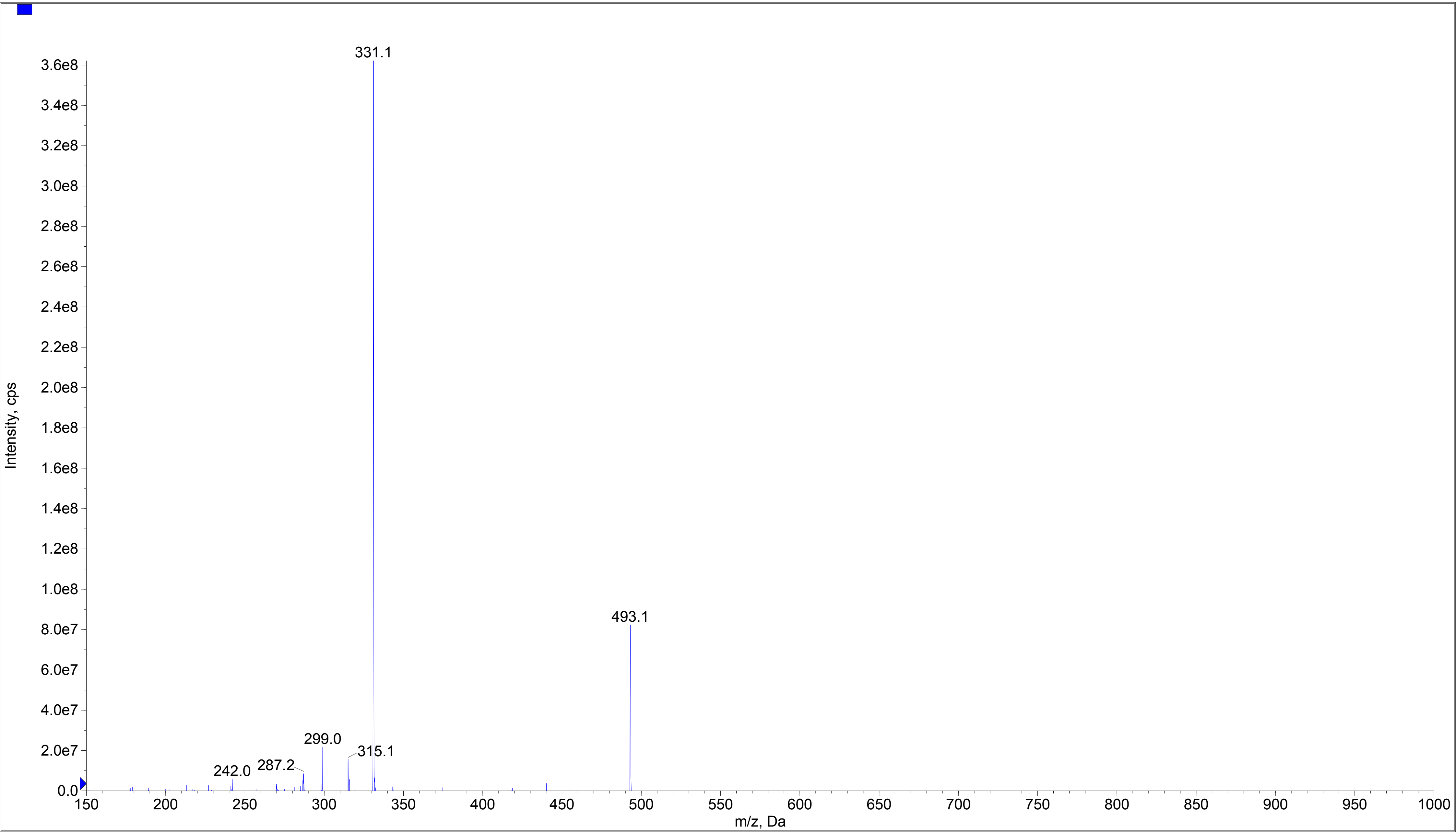

Acq. File:
Anthocy_WH6500-3_BEH-XL-1_V2.0_MHX_EPI_20210511.wiff

Sample Name: Anthocy_mix sample_5000_CE40
Sample Number: N/A

Malvidin-3-O-rutinoside

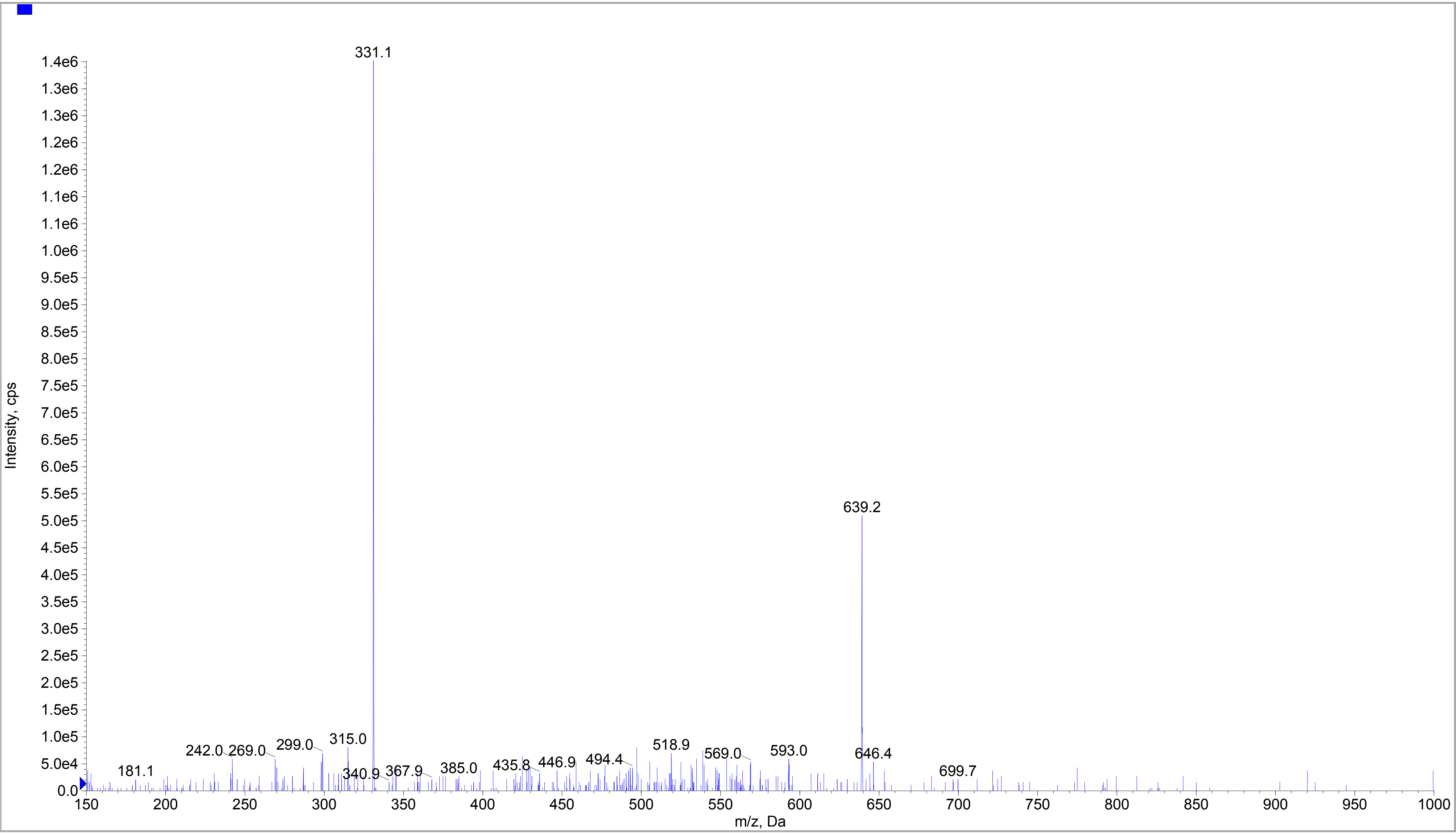

Acq. File:
Anthocy_WH6500-3_BEH-XL-1_V2.0_MHX_EPI_20210511.wiff

Sample Name: STD_5000ppb_5000_CE35
Sample Number: N/A

Pelargonidin-3,5-O-diglucoside

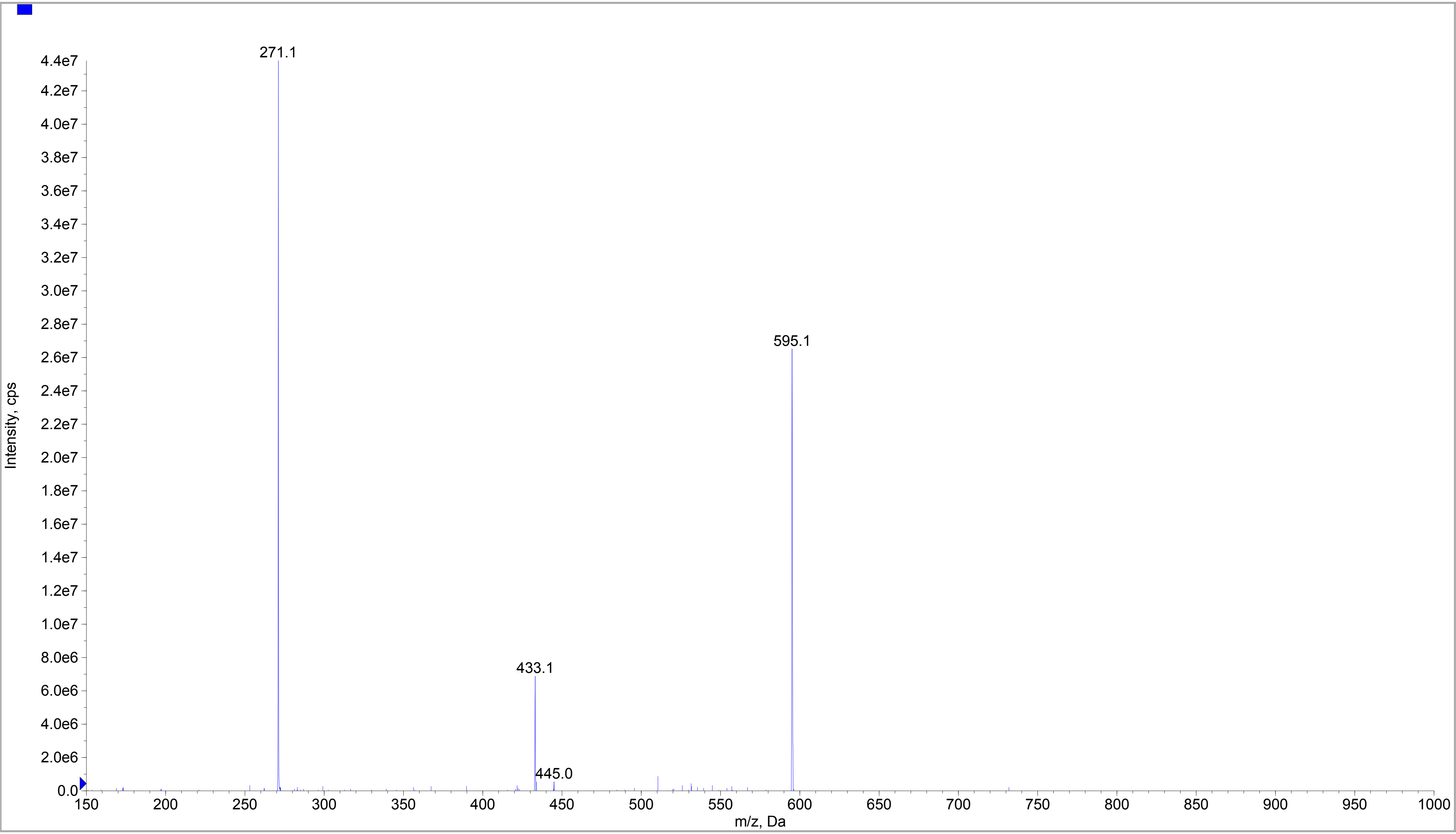

Acq. File:
Anthocy_WH6500-3_BEH-XL-1_V2.0_MHX_EPI_20210511.wiff

Sample Name: A21014992a_5000_CE40
Sample Number: N/A

Pelargonidin-3-O-(6-O-malonyl-beta-D-glucoside)

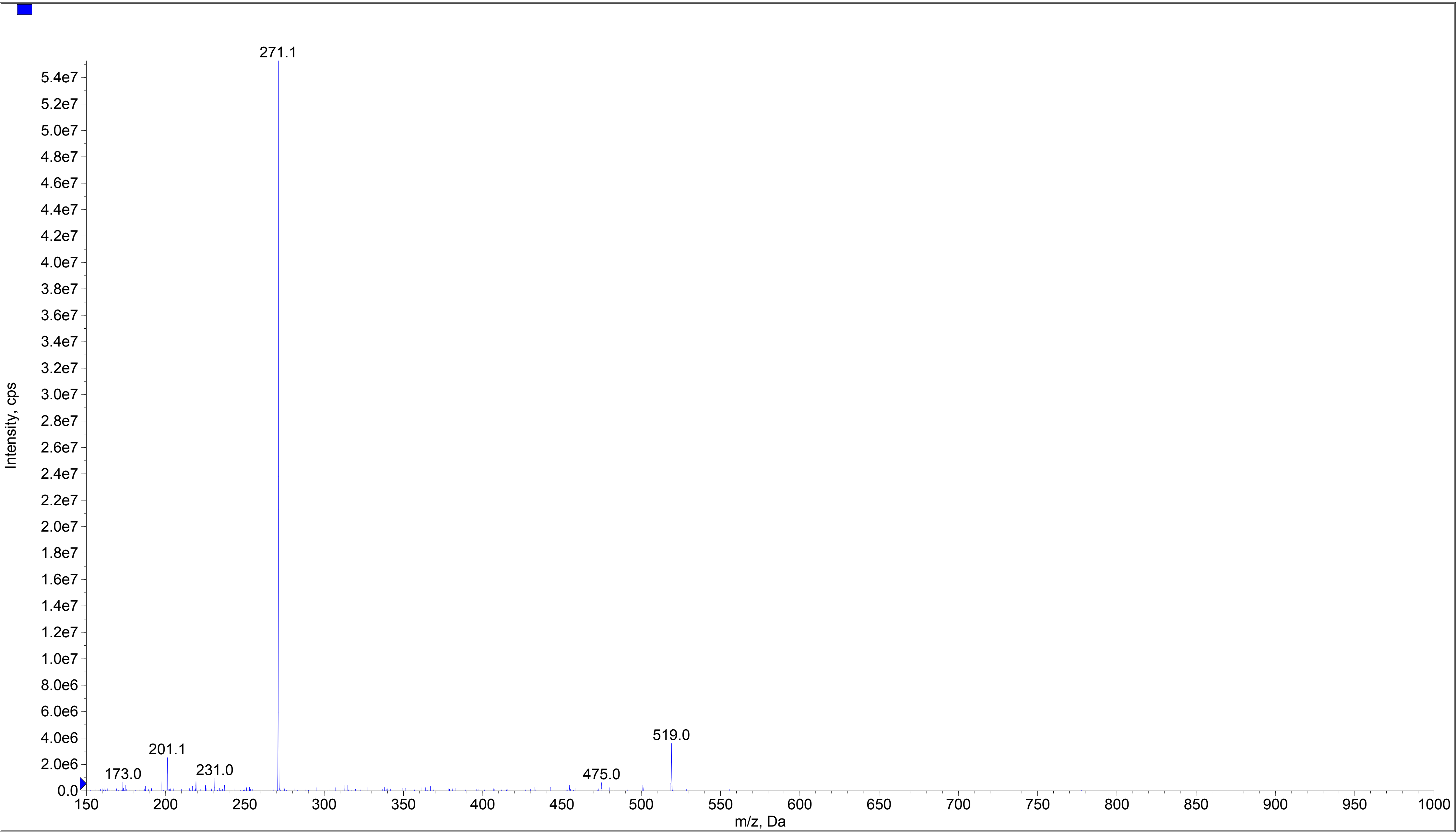

Acq. File:
Anthocy_WH6500-3_BEH-XL-1_V2.0_MHX_EPI_20210511.wiff

Sample Name: Anthocy_mix sample_5000_CE40
Sample Number: N/A

Pelargonidin-3-O-arabinoside

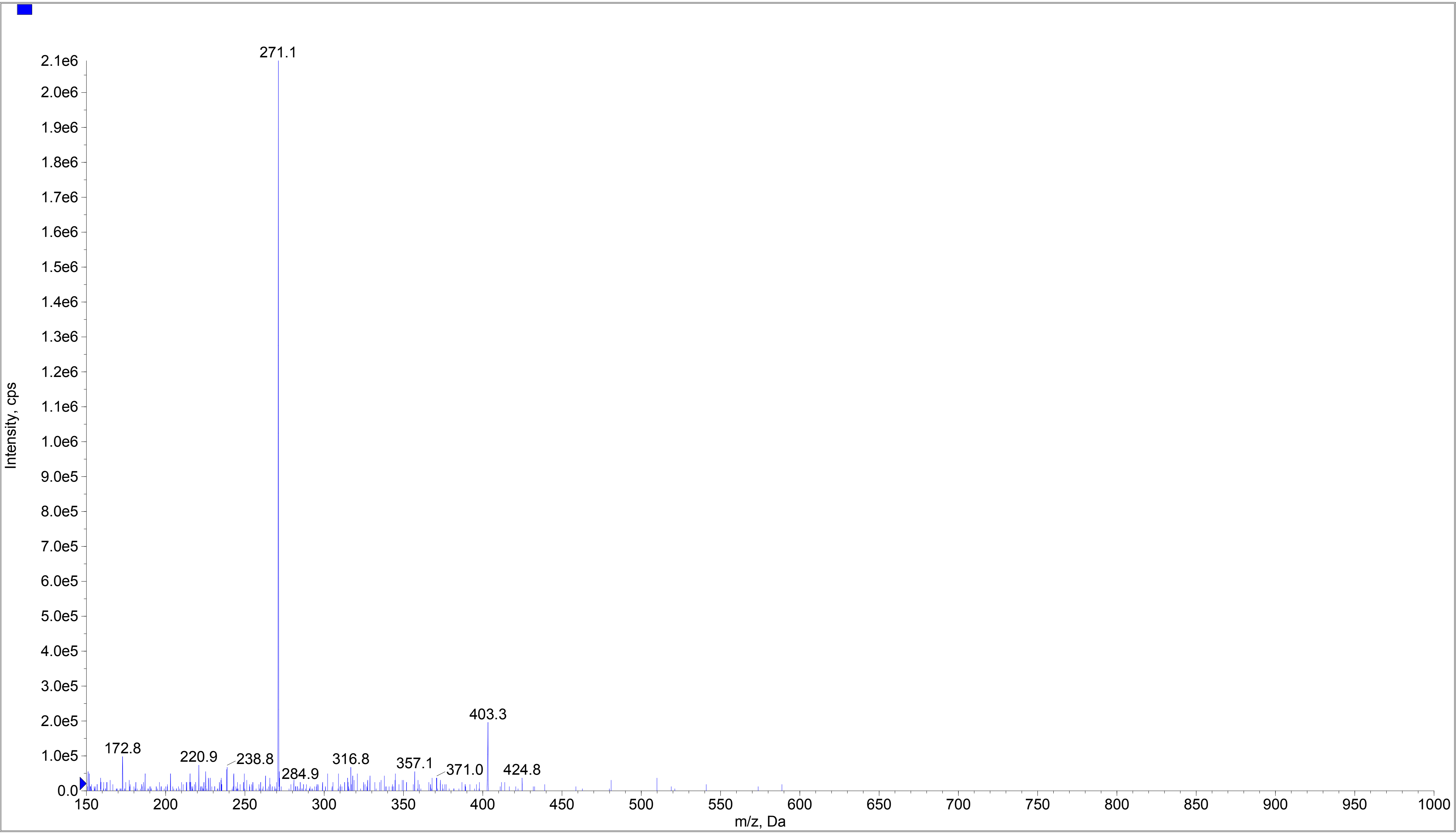

Acq. File:
Anthocy_WH6500-3_BEH-XL-1_V2.0_MHX_EPI_20210511.wiff

Sample Name: STD_5000ppb_5000_CE35
Sample Number: N/A

Pelargonidin-3-O-glucoside

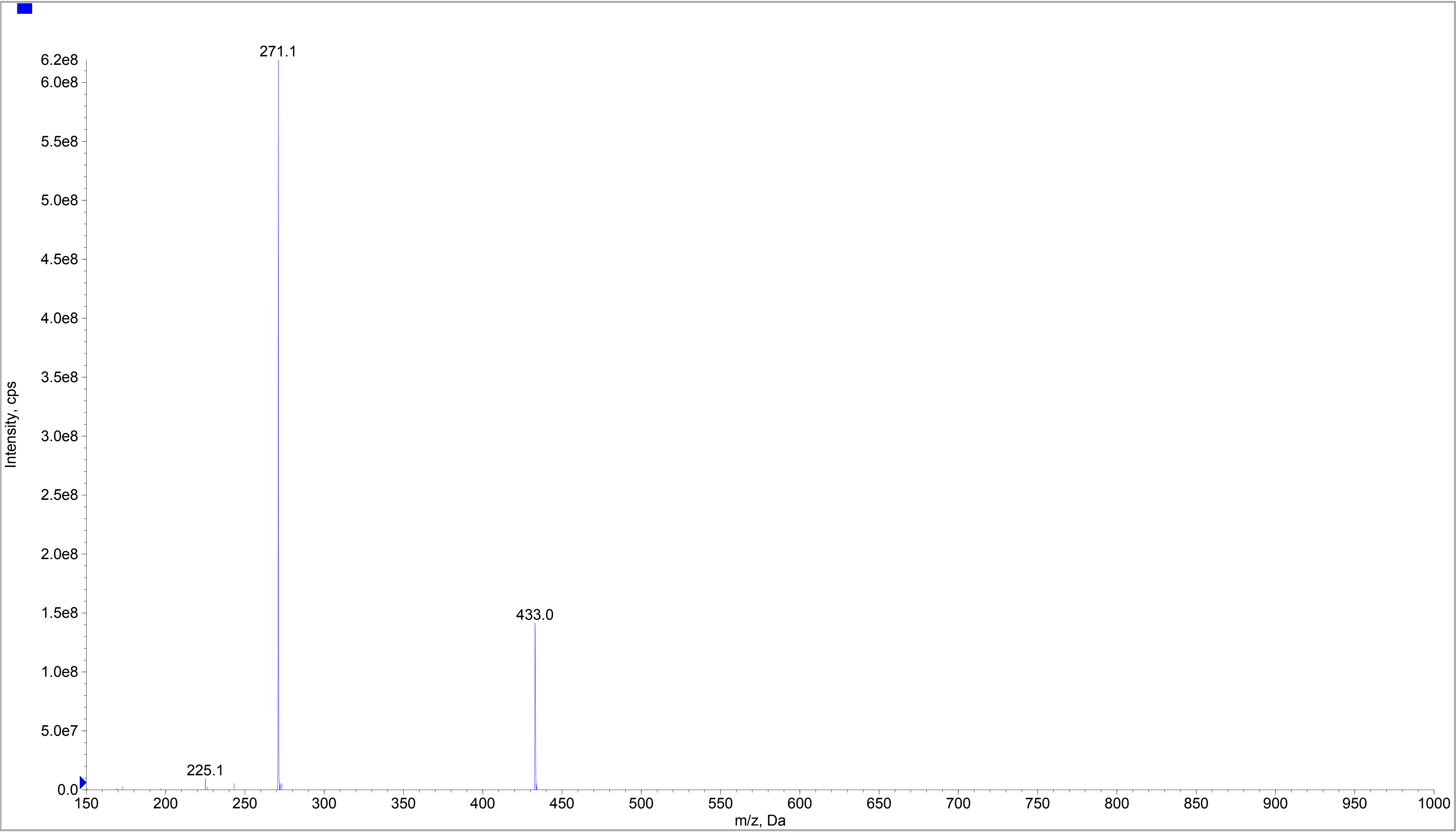

Acq. File:
Anthocy_WH6500-3_BEH-XL-1_V2.0_MHX_EPI_20210511.wiff

Sample Name: Anthocy_mix sample_5000_CE40
Sample Number: N/A

Pelargonidin-3-O-rutinoside

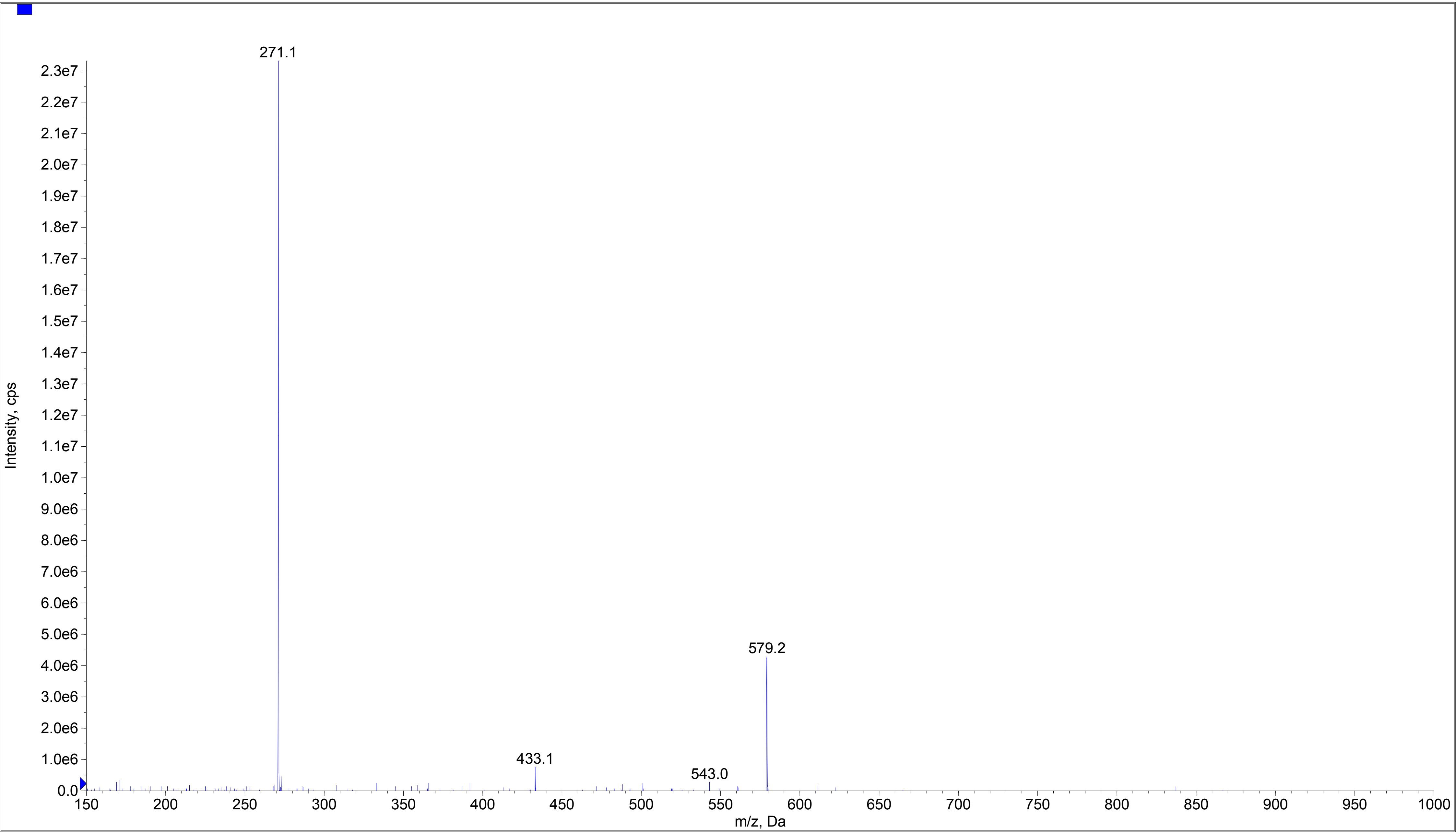

Acq. File:
Anthocy_WH6500-3_BEH-XL-1_V2.0_MHX_EPI_20210511.wiff

Sample Name: STD_5000ppb_5000_CE40
Sample Number: N/A

Peonidin-3,5-O-diglucoside

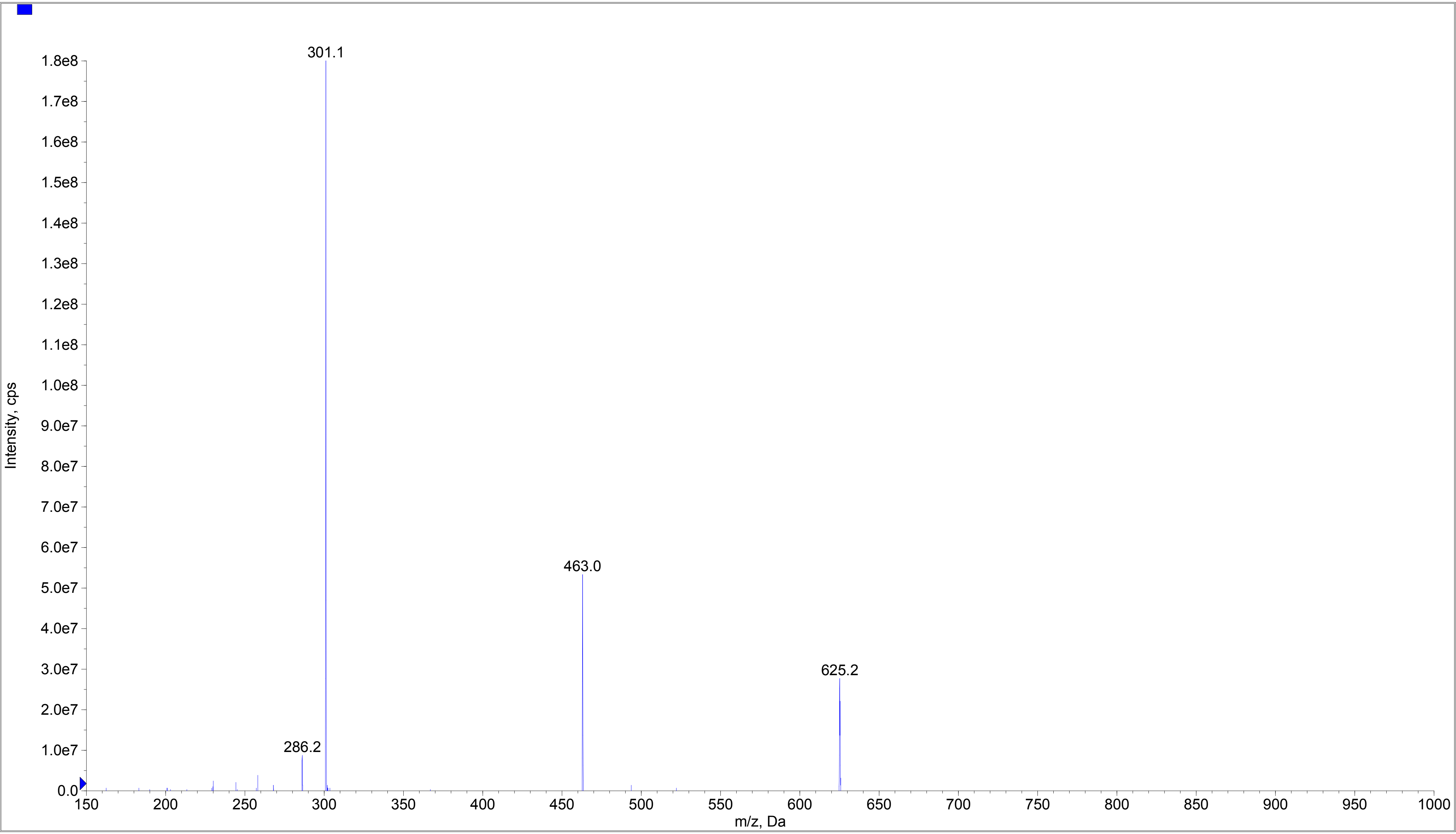

Acq. File:
Anthocy_WH6500-3_BEH-XL-1_V2.0_MHX_EPI_20210511.wiff

Sample Name: A21014992a_5000_CE40
Sample Number: N/A

Peonidin-3-O-(6-O-malonyl-beta-D-glucoside)

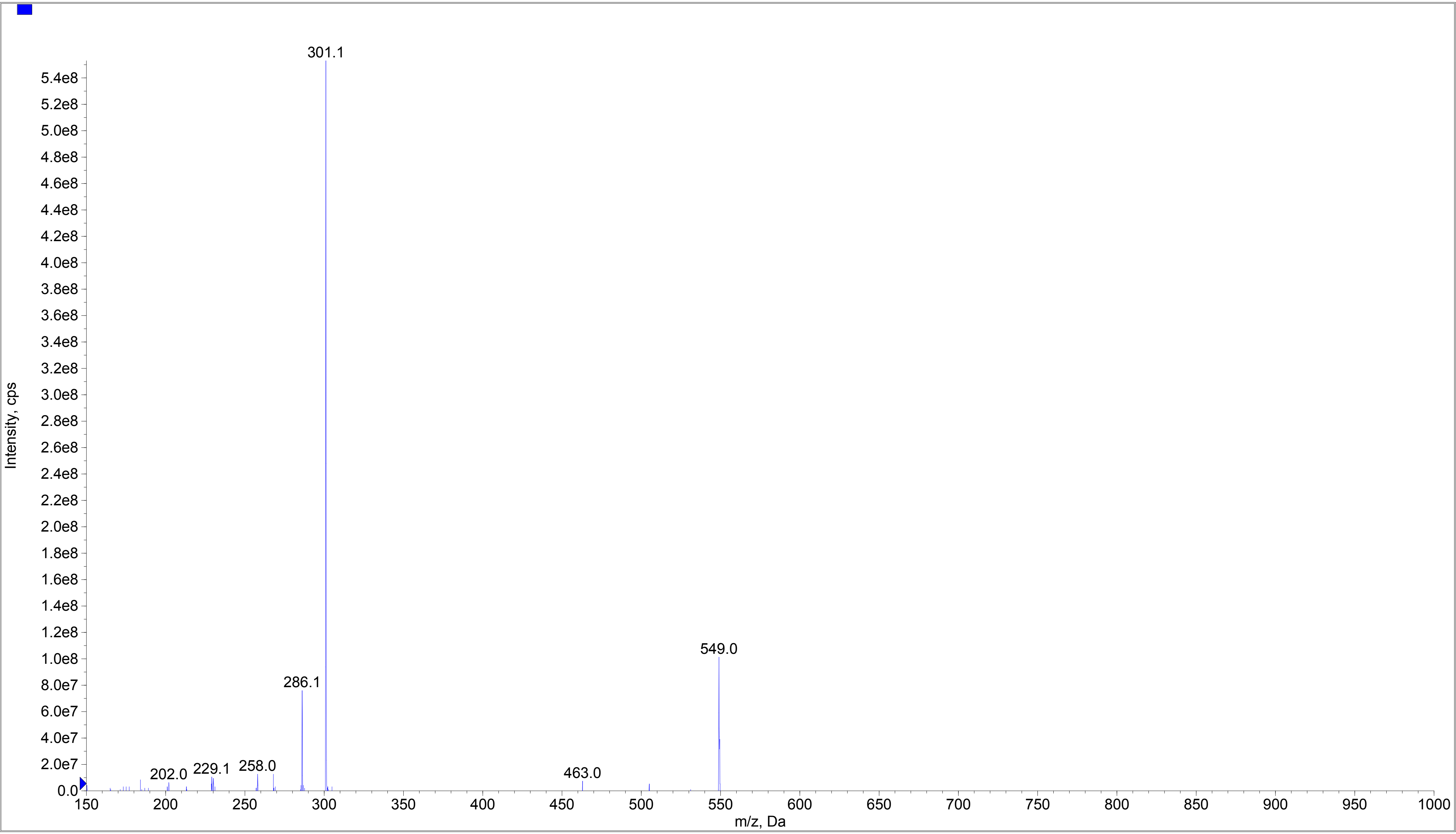

Acq. File:
Anthocy_WH6500-3_BEH-XL-1_V2.0_MHX_EPI_20210511.wiff

Sample Name: STD_2000ppb_5000_CE35
Sample Number: N/A

Peonidin-3-O-arabinoside

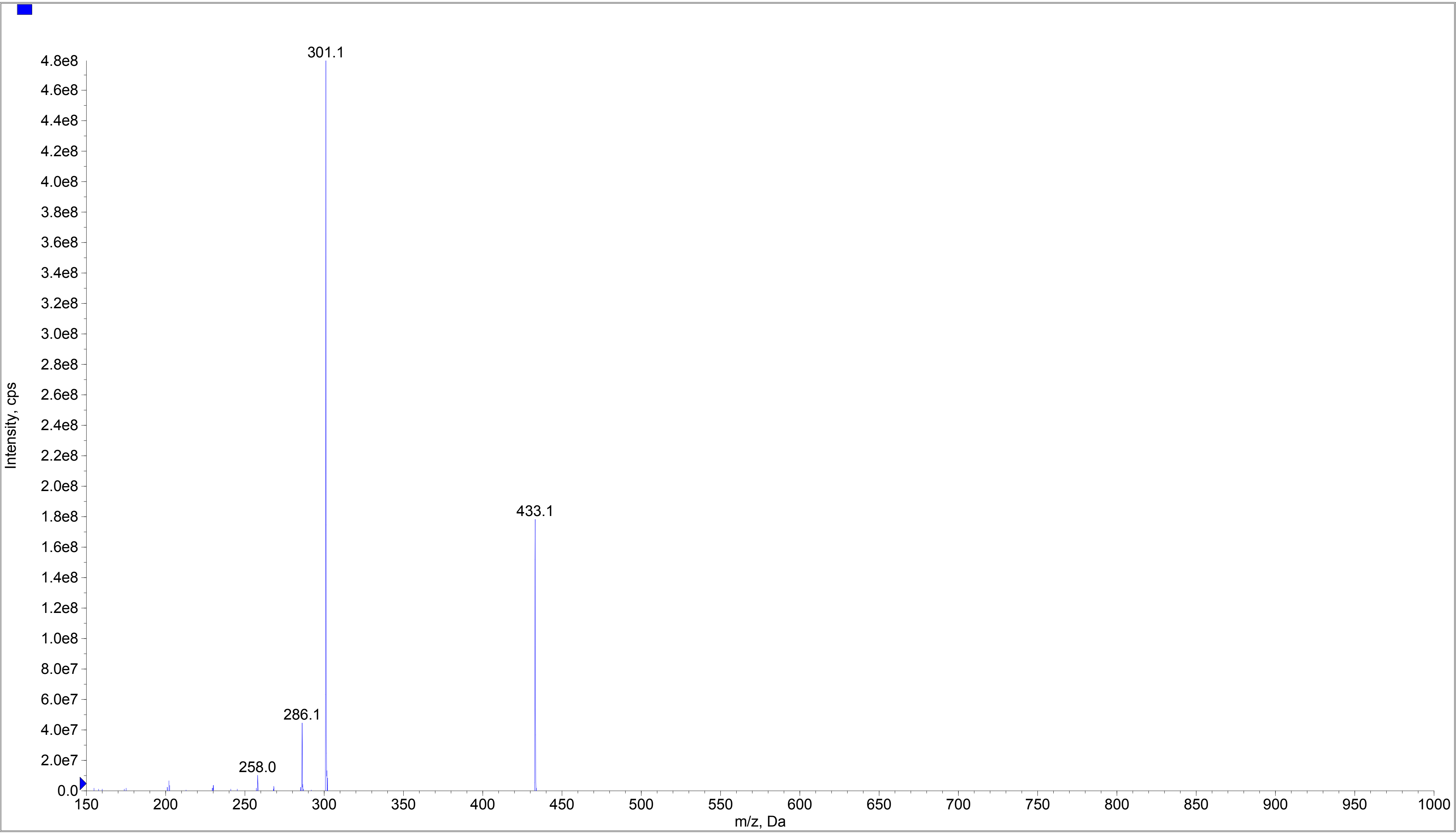

Acq. File:
Anthocy_WH6500-3_BEH-XL-1_V2.0_MHX_EPI_20210511.wiff

Sample Name: STD_2000ppb_5000_CE35
Sample Number: N/A

Peonidin-3-O-galactoside

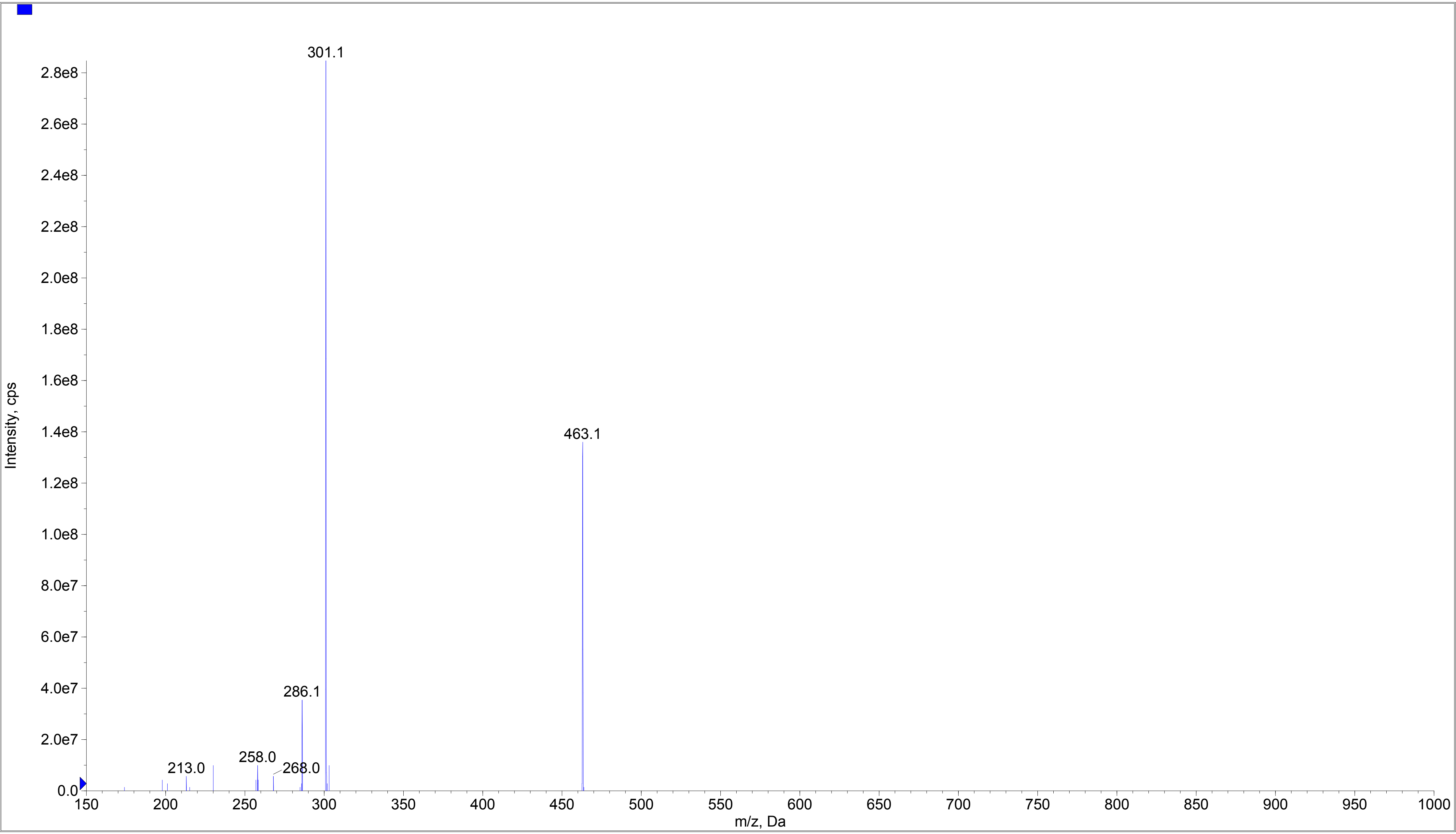

Acq. File:
Anthocy_WH6500-3_BEH-XL-1_V2.0_MHX_EPI_20210511.wiff

Sample Name: STD_5000ppb_5000_CE35
Sample Number: N/A

Peonidin-3-O-glucoside

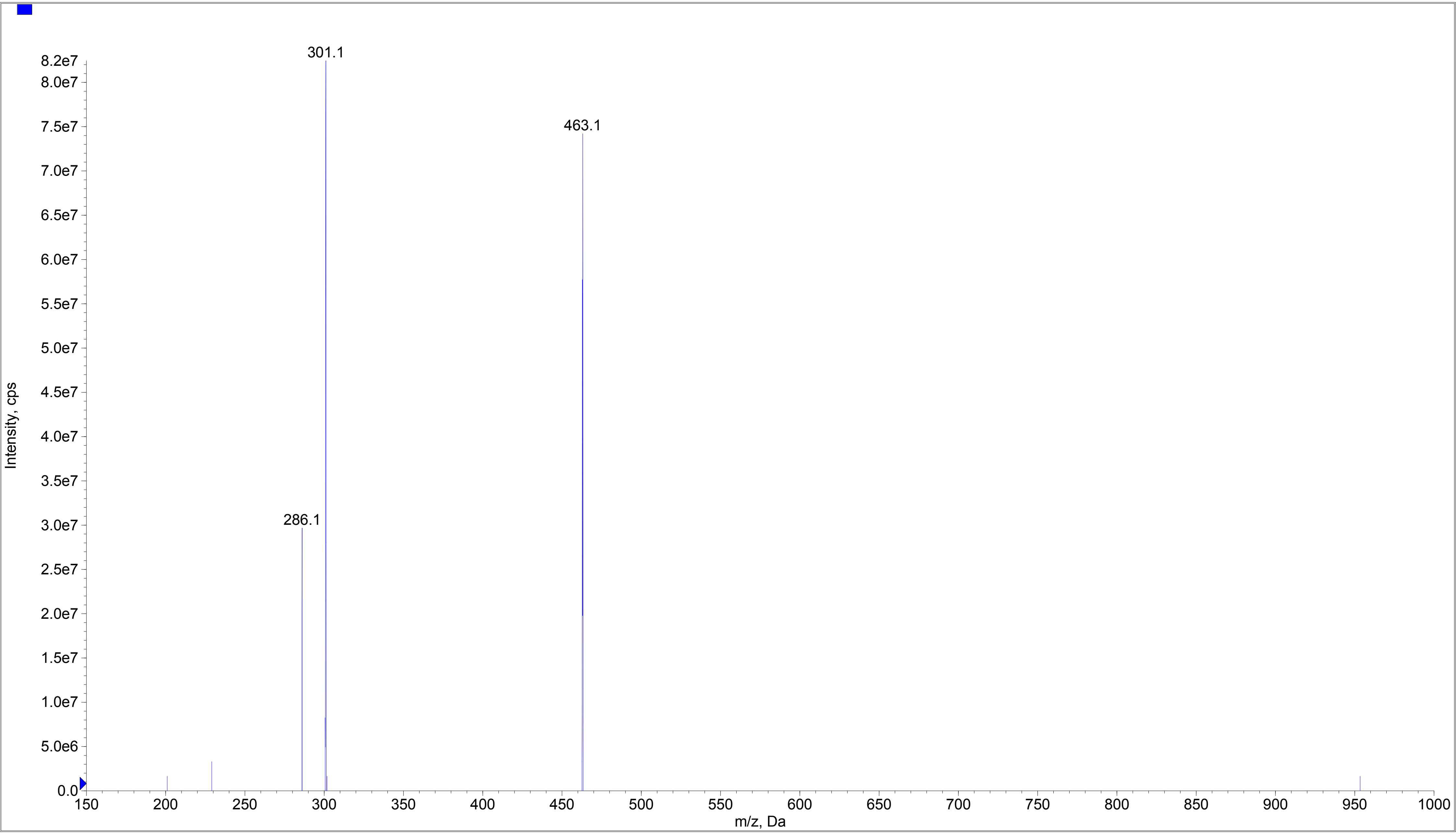

Acq. File:
Anthocy_WH6500-3_BEH-XL-1_V2.0_MHX_EPI_20210511.wiff

Sample Name: Anthocy_mix sample_5000_CE40
Sample Number: N/A

Peonidin-3-O-rutinoside

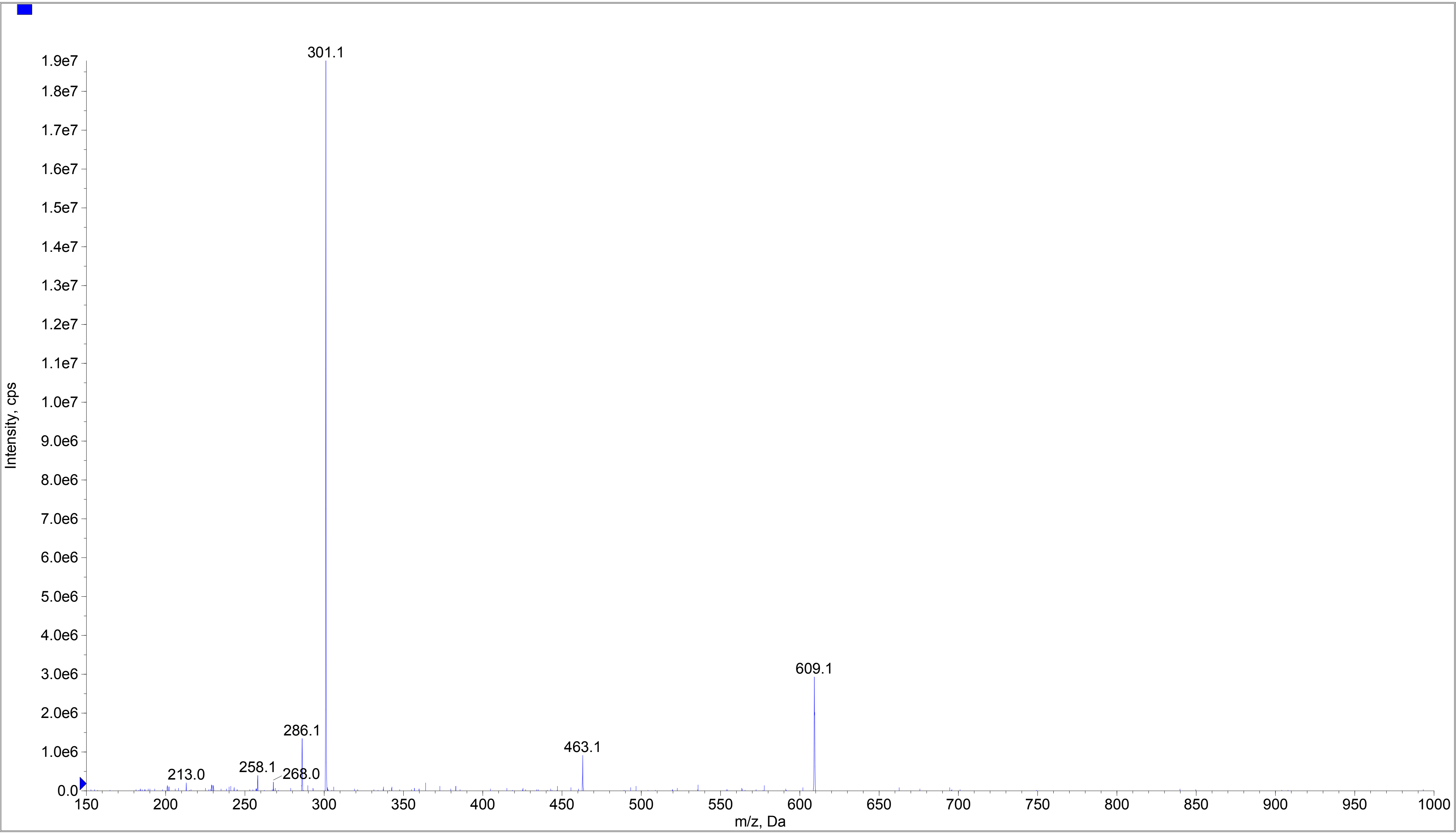

Acq. File:
Anthocy_WH6500-3_BEH-XL-1_V2.0_MHX_EPI_20210511.wiff

Sample Name: A21023223a_a_5000_CE40
Sample Number: N/A

Petunidin-3-O-(6-O-malonyl-beta-D-glucoside)

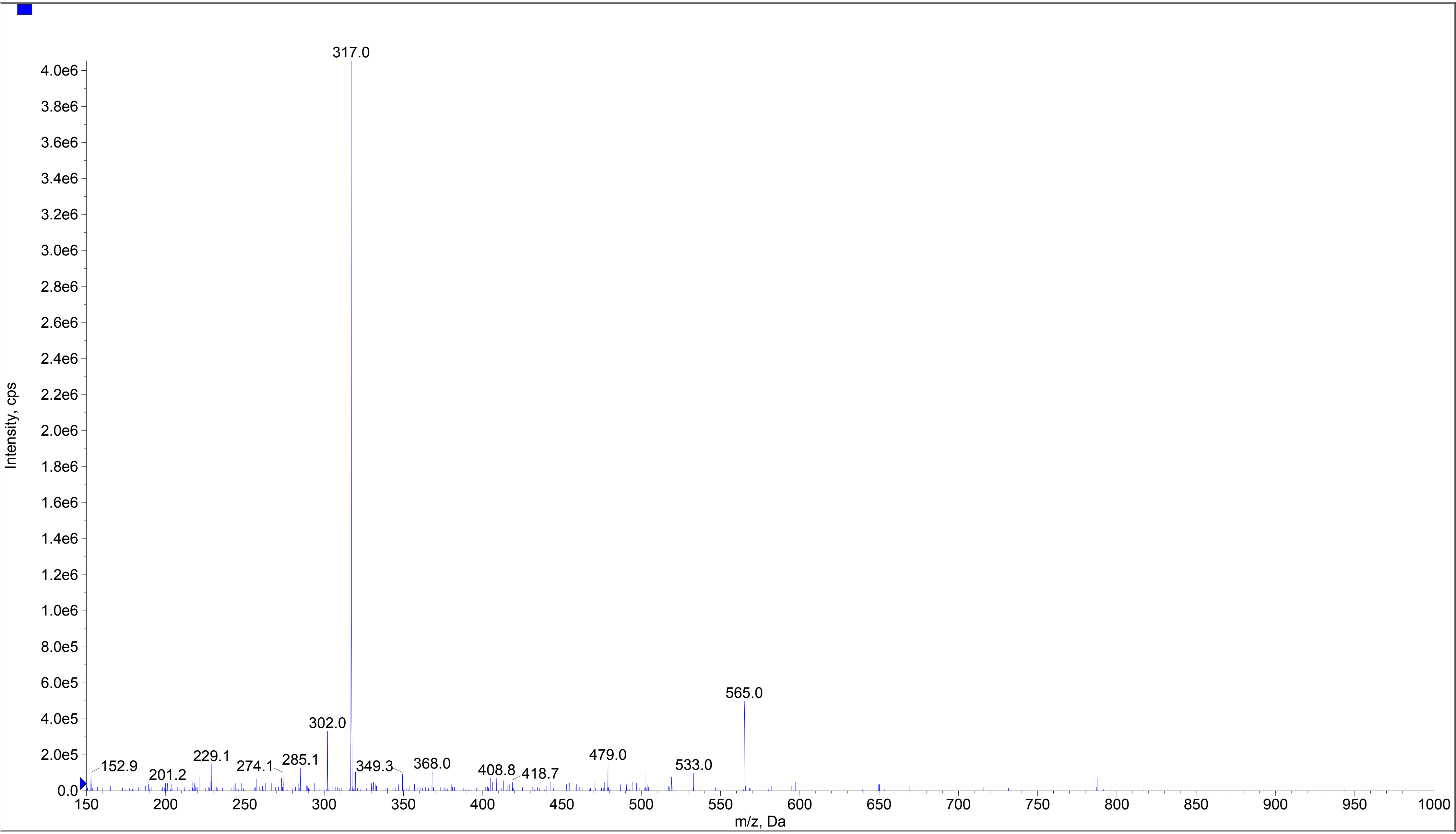

Acq. File: Anthocy_WH6500-3_BEH-XL-1_V2.0_MHX_EPI_20210511.wiff

Sample Name: Anthocy_mix sample_5000_CE40
Sample Number: N/A

Petunidin-3-O-arabinoside

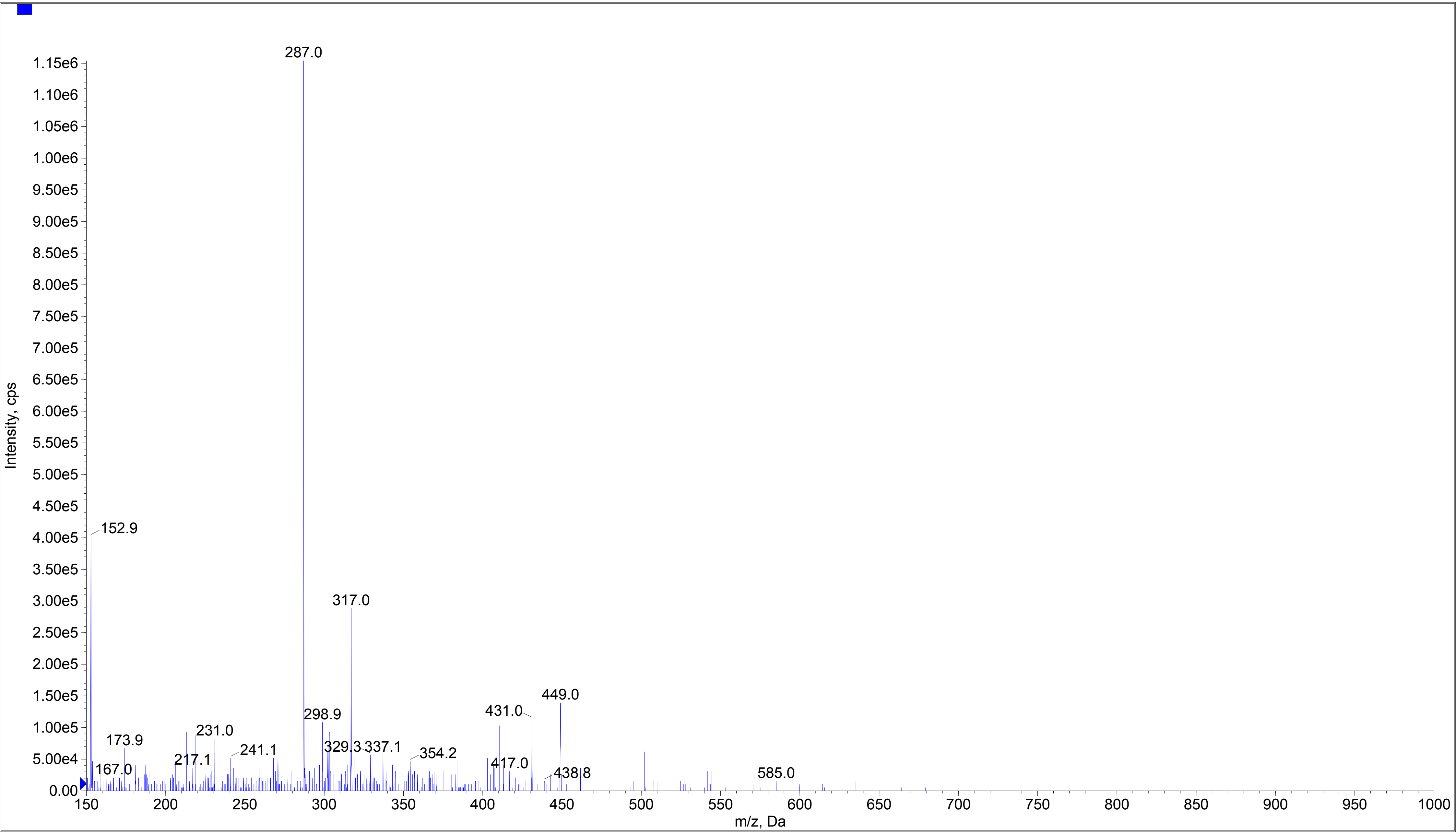

Acq. File:
Anthocy_WH6500-3_BEH-XL-1_V2.0_MHX_EPI_20210511.wiff

Sample Name: STD_2000ppb_5000_CE35
Sample Number: N/A

Petunidin-3-O-galactoside

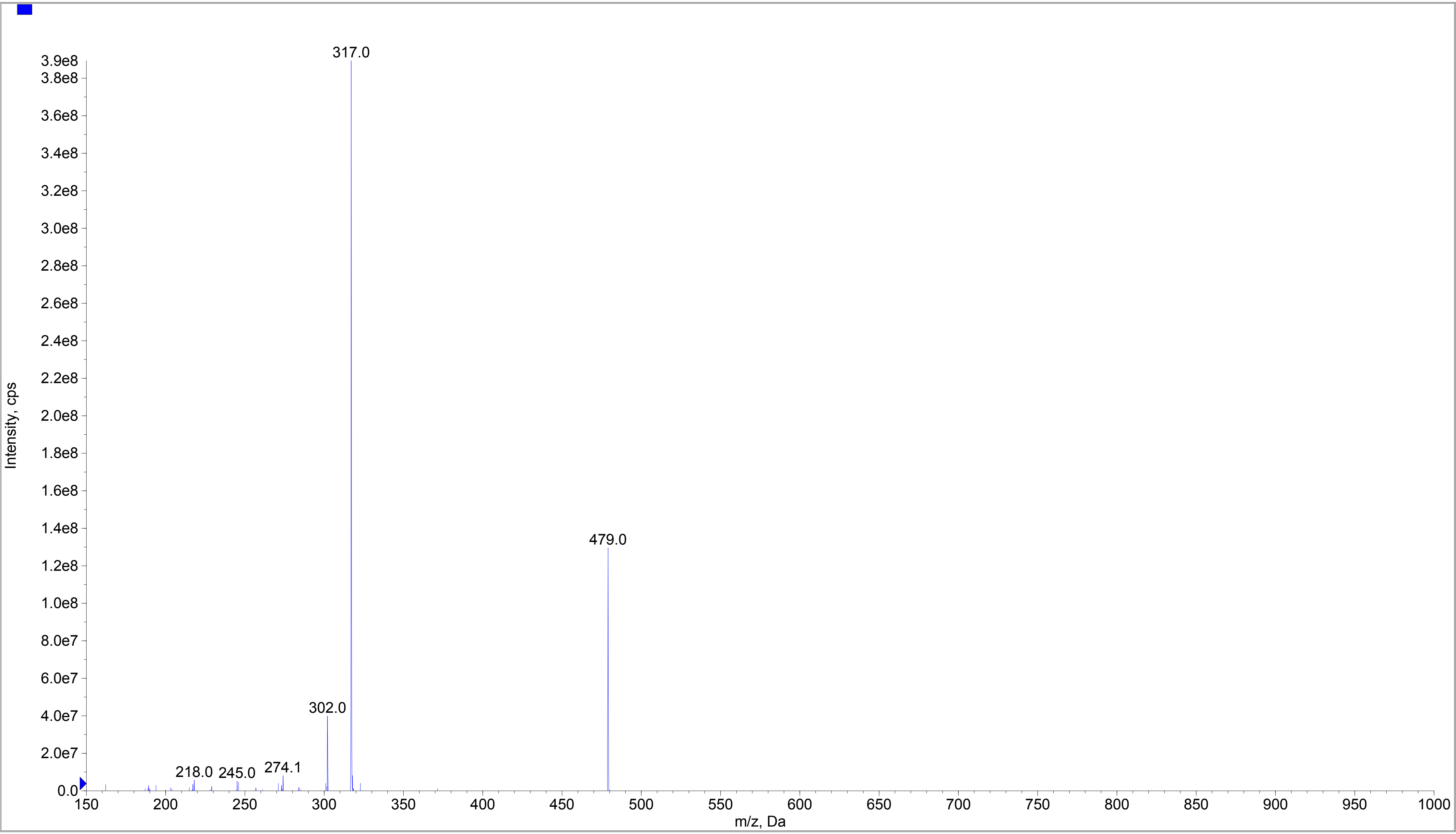

Acq. File:
Anthocy_WH6500-3_BEH-XL-1_V2.0_MHX_EPI_20210511.wiff

Sample Name: STD_5000ppb_5000_CE35
Sample Number: N/A

Petunidin-3-O-glucoside

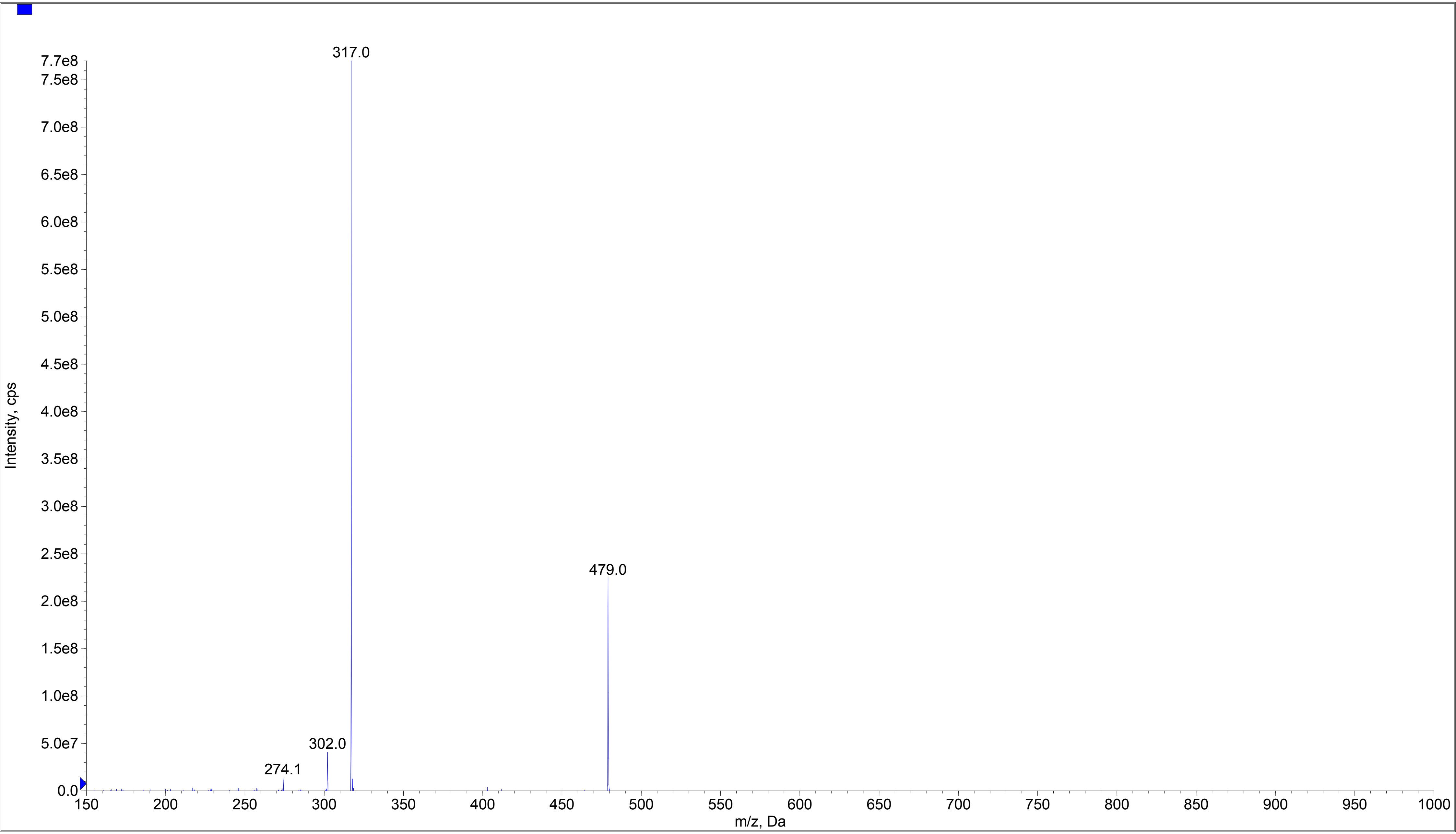

Acq. File:
Anthocy_WH6500-3_BEH-XL-1_V2.0_MHX_EPI_20210511.wiff

Sample Name: Anthocy_mix sample_5000_CE40
Sample Number: N/A

Petunidin-3-O-rutinoside

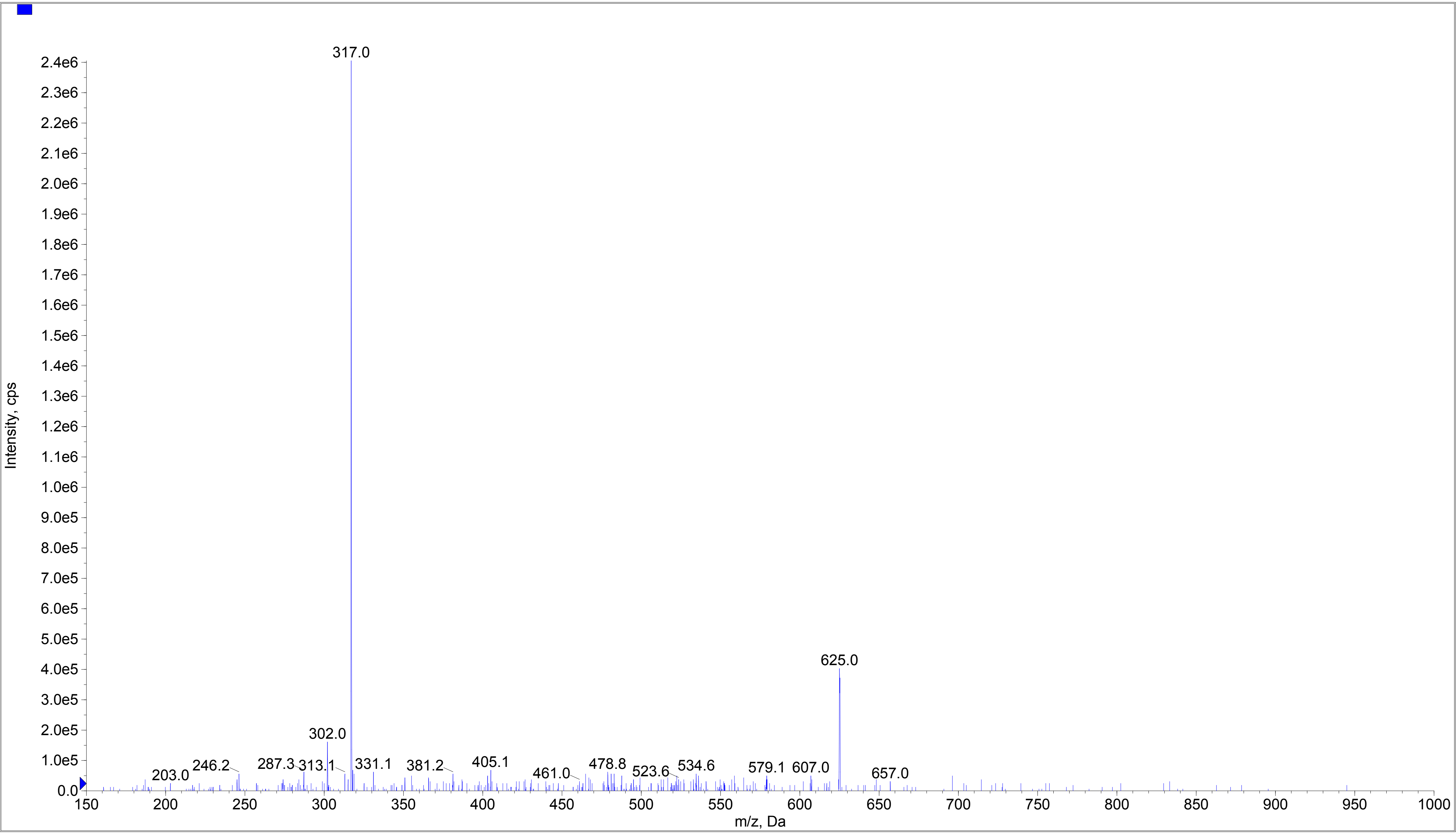

Acq. File:
Anthocy_WH6500-3_BEH-XL-1_V2.0_MHX_EPI_20210511.wiff

Sample Name: STD_5000ppb_5000_CE35
Sample Number: N/A

Quercetin-3-O-glucoside

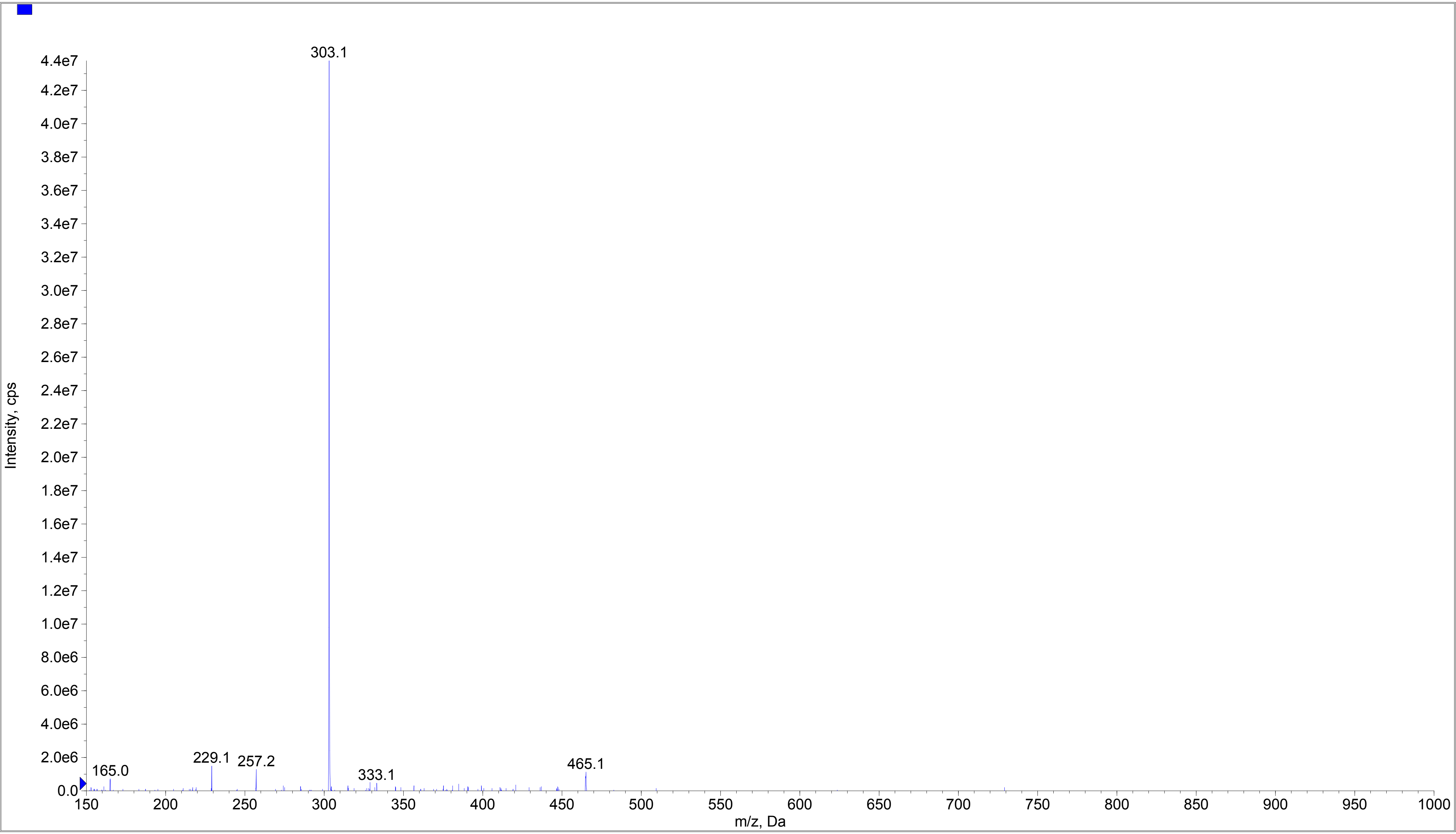

Figure S2. The chromatograms of flavonoids.
